# Agnuside from *Vitex negundo* L. Attenuates Hepatocellular Lipid Accumulation: Association with the AMPK/SREBP1/FASN/HMGCR/PPARα/UCP2 Signaling Network

**DOI:** 10.1021/acsomega.6c06583

**Published:** 2026-10-28

**Authors:** Abhipsha Saikia, Semim Akhtar Ahmed, Kangkon Saikia, Pranamika Sarma, Devi Basumatary, Ashis K. Mukherjee, Jagat Chandra Borah, Asis Bala

**Affiliations:** † Life Sciences Division, 163940 Institute of Advanced Study in Science & Technology, Guwahati, Assam 781035, India; ‡ Academy of Scientific and Innovative Research (AcSIR), Ghaziabad 201002, India; § Department of Molecular Biology and Biotechnology, Tezpur University, Tezpur, Assam 784028, India; ∥ Department of Medicinal Chemistry, National Institute of Pharmaceutical Education and Research (NIPER), Guwahati, Assam 781101, India

## Abstract

*Vitex negundo* L. (VN) is a medicinal plant known for its anti-inflammatory and hepatoprotective properties, commonly used as a vegetable in northeastern Indian communities. However, its role in treating nonalcoholic fatty liver disease (NAFLD) is still underexplored. This study evaluates the therapeutic potential of VN and its bioactive compounds in reducing lipid accumulation in CC1 hepatocytes. A crude extract of VN and its *n*-butanol fraction (VNBU) were tested on palmitate-induced CC1 hepatocytes to evaluate lipid accumulation. Key compounds Agnuside, 5-hydroxy-3,6,7,3́,4́-pentamethoxyflavone, and Vitexilactone were isolated. Agnuside’s effects on lipogenesis, cholesterol accumulation, and fatty acid oxidation were assessed using biochemical assays and Western blot analysis. Molecular docking and dynamics simulations were conducted to explore its interactions with 3-hydroxy-3-methylglutaryl-coenzyme A reductase (HMGCR). Agnuside treatment was associated with reduced lipid accumulation by modulating Adenosine monophosphate-activated protein kinase (AMPK), HMGCR, Sterol regulatory element-binding transcription factor 1 (SREBP1), Fatty acid synthase (FASN), Peroxisome Proliferator-activated receptors (PPARα), and uncoupling protein (UCP2), suggesting a favorable shift in lipid metabolism. Computational studies indicated that Agnuside binds stably to HMGCR, similar to statins. In summary, these findings suggest that VN and Agnuside exert protective effects in hepatocytes, accompanied by favorable shifts in lipid metabolism markers. Further studies are needed to confirm the causal role of this signaling network.

## Graphical abstract

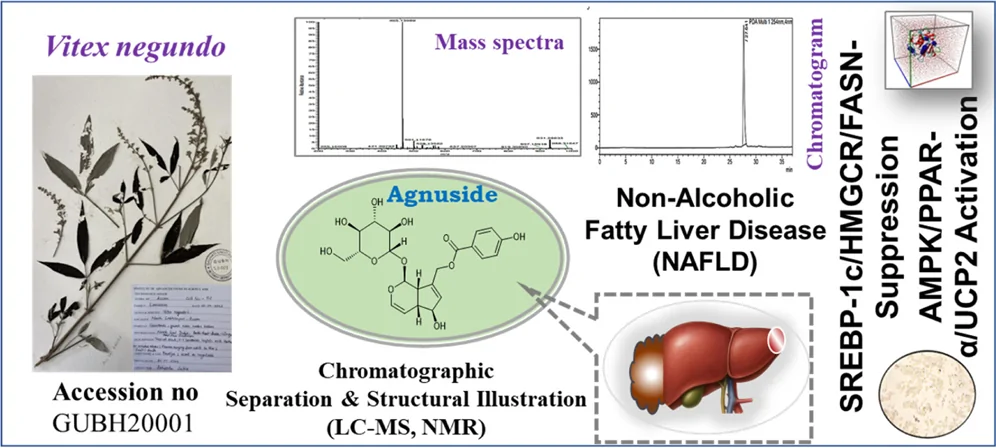

## 1 Introduction

Hepatic steatosis, or fatty liver disease, is characterized by the excessive buildup of triglycerides within hepatocytes due to lipid metabolism and storage imbalances. The liver, a key organ for detoxification, plays a central role in lipid homeostasis through fat synthesis, metabolism, transport, and storage. Disruptions in these processes contribute to the onset of nonalcoholic fatty liver (NAFLD), a rising global health issue affecting about 25% of the population, 13% in Africa and up to 42% in Southeast Asia.^1^ In developed countries, NAFLD is a major health burden, progressing from simple steatosis to nonalcoholic steatohepatitis, which involves hepatocyte damage, chronic inflammation, and fibrosis.^2^ NAFLD, now referred to as MASLD in current international consensus nomenclature, represents the hepatic manifestation of systemic metabolic dysfunction and is closely linked to obesity, insulin resistance, dyslipidemia, and cardiovascular risk.^3,4^

The pathogenesis of NAFLD is primarily driven by excessive hepatic lipid accumulation caused by increased free fatty acid influx, impaired β-oxidation, and enhanced *de novo* lipogenesis.^5^ Since there is no specific pharmacological treatment currently exists, NAFLD management depends largely on lifestyle modifications, including dietary improvements and increased physical activity.^6^ A key mechanism contributing to hepatic lipid accumulation involves SREBP-1c and FASN, major lipogenesis regulators. AMPK, a critical energy-sensing enzyme, modulates their activity. Studies have shown that the SREBP-1c/FASN pathway is upregulated in NAFLD models, contributing to disease progression.^7^ Inhibition of SREBP-1c has been reported to reduce hepatic steatosis in diet-induced obese mice,^8^ and a strong link has been established between downregulation of this pathway and decreased hepatic lipid synthesis. Additionally, uncoupling protein 2 (UCP2), a mitochondrial protein that regulates energy balance and oxidative stress, improves mitochondrial function and enhances fatty acid oxidation. The activation of the AMPK/PPAR-α/UCP2 pathway not only boosts lipid catabolism but also reduces oxidative stress, a key contributor to steatohepatitis. Thus, modulating these molecular targets, activating AMPK/PPAR-α/UCP2 and inhibiting SREBP-1c/FAS, offers promising therapeutic avenues for preventing hepatic lipid accumulation and managing NAFLD. Building on the urgent need for effective treatments beyond lifestyle modifications for NAFLD, natural products have garnered renewed interest due to their therapeutic potential. Many plant-derived bioactive phytoconstituents possess protective effects against various diseases, although some remain uncharacterized despite traditional use and clinical interest.^9–11^ Among such plants, *Vitex negundo* L. (VN), the five-leaf chaste tree or *nirgundi* in India specially northeast region, has been traditionally used for medicinal and edible purposes. VN leaves and seeds are typically consumed as condiments, teas, and functional foods.^12,13^ Also, its leaves have high nutritional value. The previous analysis shows that the moisture content is 82.78 ± 2.68*g*/100 g DW, protein content is 19.27 ± 0.85*g*/100 g DW, fat content is 3.13 ± 0.51*g*/100 g DW, fiber content is 19.70 ± 0.90*g*/100 g DW, and carbohydrate content is 37.75 ± 0.75*g*/100 g DW. The previous data indicate the presence of micro minerals (Na, K, Mg, Ca) and macro minerals (Fe, Zn, Cu).^14^ The leaves were consumed by people for their anti-inflammatory and health-promoting effects. VN exhibits diverse pharmacological properties, including antioxidant, antimicrobial, anti-inflammatory, anticancer, hypouricemic, and CNS-depressant activities. Agnuside (AGN), a key bioactive compound from VN, has shown potential in treating inflammatory conditions, oxidative stress, metabolic disorders, and microbial infections.^15–18^

Notwithstanding its widespread traditional and pharmaceutical applications, there is now a deficiency of studies investigating the impact of VN or AGN on lipid metabolism and hepatic steatosis, especially concerning NAFLD. This work aims to examine the potential therapeutic effects of crude and its active component AGN in a palmitic acid (PA)-induced *in vitro* model of nonalcoholic fatty liver disease (NAFLD) utilizing CC1 hepatocytes. The research also investigated the underlying mechanism of action of AGN, bolstered by in silico analysis, to establish a solid foundation for its hepatoprotective potential.

## 2 Materials and Methods

### 2.1 Materials

Bruker Avance neo 400 MHz NMR instrument was used to record the NMR spectra. HRP-linked secondary antimouse and antirabbit antibodies, AMPKα, pAMPK-α (Thr172) (40H9), were acquired from Cell Signaling Technology, Beverly, Massachusetts, USA. Anti-SREBP1 antibodies were obtained from Santa Cruz Biotech, Dallas, Texas, USA. Novus Biologicals, Englewood, USA, supplied anti-HMGCR, antiphosphorylated HMGCR (Ser 872), anti-FAS (#ab22759), and anti-PPARα (#ab8934), which were procured from Abcam, Waltham, MA, USA. Merck (Darmstadt, Germany) provided primary antibody anti-UCP2 (#662047). Antibiotic antimycotic, Trypsin, FBS and DPBS were purchased from Gibco, Waltham, MA, USA. Thermo Scientific, Waltham, MA, USA, provided phosphate inhibitor, PMSF and Halt protease. Sigma-Aldrich Co., St. Louis, MO, USA, supplied Sodium palmitate, BSA, Acrylamide, Triton X, Bis acrylamide, and RIPA buffer. SGOT kit and SGPT kit were procured from Coral. From Sigma-Aldrich, other necessary reagents and chemicals were acquired.

### 2.2 Plant Sample Collection, Extraction and Fractionation

Fresh VN leaves were procured from North Lakhimpur, Assam, India. The specimen was verified at the Department of Botany, Guwahati University, Guwahati, India, under accession number GUBH20001. The taxonomically accepted name was verified from the Web site ″The WFO Plant List″ (https://wfoplantlist.org).

VN leaves were washed, shed-dried, and ground into a powder form. A 500 g sample was subjected to cold maceration with 3 L of methanol at room temperature for 24 h, repeated thrice. The methanolic extracts were concentrated via rotary evaporation at 45 °C to produce a crude extract. Solvent–solvent fractionation of the crude extract was carried out based on increasing polarity of the solvents to obtain four fractions as *n*-hexane, ethyl acetate, *n*-butanol and water fraction. From 90 g of crude extract, solvent–solvent fractionation yielded 21 g of hexane fraction, 14 g of ethyl acetate fraction, 18 g of *n*-butanol Fraction, and 23 g of water Fraction, each of which was subsequently evaporated at 45 °C.

### 2.3 HPLC-DAD Analysis of VN Extract, Fractions and its Active isolated Compound

The extract and fractions were analyzed using high-performance liquid chromatography (HPLC). For sample preparation, each sample was dissolved in a 1:1 *v/v* solution of water and methanol and filtered through a 0.45 μm Millex-HN syringe filter before being injected into a Shimadzu Shim-packed GIS C18 analytical column (5 μm, 4.6 × 250 mm) at a flow rate of 1 mL/min. Injection volumes of 20 μL for the analytical sample were taken for the analysis. The mobile phase consisted of solvent A (0.1% Trifluoroacetic acid in water) and solvent B (methanol), using a linear gradient over a total run time of 37 min. The column was equilibrated with 100% solvent A (0.1% TFA in water). The linear gradient started at 5 min, reaching 100% of solvent B (methanol) at 30 min. This condition was maintained for 2 min before reaching the initial column condition at 35 min. The column was allowed to contain the initial condition for two more minutes before the run time stopped at 37 min. HPLC spectra were recorded in a Shimadzu Prominence Binary Gradient HPLC system LC20AP with PDA detector SPD-M20A.

### 2.4 Isolation of Bioactive Compounds from the Active Fraction

A bioactivity study of the different fractions prepared from the VN extract resulted in the *n*-butanol fraction being the most active. Thus, phytochemical isolation of the most active fraction was carried out to identify the bioactive constituents. Column chromatography of the *n*-butanol fraction (14 g) was performed using HP-20 Diaion resin with methanol and water as eluting solvents. The required fraction was dissolved with 100% water, and the concentration of methanol was increased gradually to obtain five subfractions: SF1­(100% water), SF2­(25% methanol), SF3 (50% methanol), SF4 (75% methanol), and SF5­(100% methanol). Three phytocompounds were isolated from the subfractions with the help of semipreparative HPLC. Isolated compounds were identified by using spectroscopic techniques.

### 2.5 Identification of Active Compounds by LCMS and FT-IR (Fourier Transform Infrared Spectroscopy) Analysis

Liquid chromatography-tandem mass spectrometry (LC–MS/MS) analysis was performed using a Thermo Scientific Ultimate 3000 LC system coupled to a Q Exactive Plus hybrid quadrupole-Orbitrap mass spectrometer (Thermo Fisher Scientific, Vienna, Austria) equipped with an electrospray ionization (ESI) source. Samples were prepared in methanol (1 mg/mL) and analyzed in both positive and negative ionization modes over an *m*/*z* range of 50–750. Data were acquired at a resolving power of 70,000 with an automatic gain control (AGC) target of 1 × 10^6^. A 10 μL aliquot of each sample was injected into the LC–MS/MS system, and the acquired mass spectra were processed using Xcalibur software.

The sample, prepared by mixing the isolated compound with KBr powder, was analyzed for its functional groups using a PerkinElmer FTIR spectrometer. The analysis was conducted at 64 scans over a wavelength range of 4000–400 cm^–1^.

### 2.6 Nuclear Magnetic Resonance (NMR)

NMR spectra of the isolated compounds were recorded on a Bruker Avance 400 MHz instrument. The isolated compounds were dissolved in DMSO­(*d*_6_) for NMR recording. ^1^H and ^13^C NMR spectra and the chemical shifts expressed in δ ppm were recorded.

### 2.7 HPLC-Assisted Quantification of Isolated Active Marker Compounds in the Crude and Active Fraction

A bioactivity study of the isolated compounds revealed that compound 1 was the most active. Thus, the crude plant extract and the active fraction were standardized to include the most-active compound. The standard solution of compound 1 was prepared in a stock concentration of 1 mg/mL and then diluted to obtain the required concentrations. Using the same standardized methods used to analyze the plant extract and the fractions, HPLC chromatograms were recorded for different concentrations of the pure compound 1, and area vs concentration standard calibration curves were constructed. The standard straight-line formula was used to estimate the active compound concentration in the extract and the active fraction.

### 2.8 Cell Culture Studies

CC1 cell line was obtained from Sigma-Aldrich catalogue No. 93070901–1VL, and the cells were preserved in a humidified incubator with 5% CO_2_ atmosphere at 37 °C in DMEM medium carrying 5.5 mM glucose, 1% antibiotic and antimycotic solution, 12 mM sodium bicarbonate 10% (v/v) fetal bovine serum (FBS).

### 2.9 Preparation of Palmitate-Conjugated BSA Solution

Sodium palmitate, conjugated with fatty acid-free bovine serum albumin, was used to treat CC1 hepatocyte cells. To prepare BSA-conjugated palmitate, sodium palmitate was dissolved in 50% (v/v) ethanol diluted in DMEM containing 2% (w/v) BSA. The solution was then incubated at 37 °C for 2 h with continuous stirring (6:1 molar ratio of palmitate: BSA, which is close to the ratio observed in human serum).

### 2.10 Treatment of Hepatocytes with Palmitate, Crude Extract and its Fractions, and Agnuside

Palmitate (PA, 0.75 mM) was added to CC1 cells with or without Crude, its fractions EAF, BUF, WF, and isolated compound C1 AGN. A positive control was employed simvastatin (Statin), a standard cholesterol-lowering drug.^19^ Various concentrations of CRUDE, EAF, BUF, WF­(1,3,5,10,20.25.50 μg/mL) and isolated compound AGN (6, 11, 21, 43, 53, 105 μM) were supplemented for 2 h followed by PA exposure for the next 20 h.

### 2.11 Cell Viability Assay

Cell viability was measured with the Alamar Blue assay. In 96-well plates, 1 × 104 cells well^–1^ were pretreated for 2 h with CRUDE until 100 μg mL^–1^ AGN until 105 μM, then challenged with 0.75 mM FFA for 20 h. After replacing the medium with Alamar Blue, plates were incubated 4 h, and absorbance was read at 570 and 600 nm. Viability (%) was calculated as [(*A*_1_ – *A*_2_)/(*P*_1_ – *P*_2_)] × 100, where *A*_1_/*A*_2_ and *P*_1_/*P*_2_ are the 570/600 nm readings of treated and positive-growth control wells, respectively.^20^

### 2.12 Triglyceride Assay

To quantify triglyceride (TG), the CC1 cells were treated with the respective drug and then washed with PBS, after which it was resuspended in 1% Triton X-100. By ultrasonication for 10 to 20 s, homogeneity of the cell suspension was maintained, followed by slow heating in the water bath for 5–7 min at around 70–100 °C. The cells were then centrifuged for 3 min to separate the nonsoluble material after the suspension was cooled to room temperature. The analysis was carried out by using a commercially available triglyceride kit (Accurex Biomedical PVT Ltd., LOT-L39022).^21^

### 2.13 Staining with Oil Red O

Oil Red O is a lipid-soluble dye that selectively binds to neutral triglycerides and lipids and stains them with a red or reddish-orange color. Oil Red O staining is conducted to reveal and identify neutral lipids (e.g., triglycerides and cholesterol esters) in the cells. The CC1 cells were fixed with 4% paraformaldehyde in phosphate buffer saline overnight, followed by 10 min of staining at room temperature with purified Oil Red O solution (0.2% in 60% isopropanol). Cells were thoroughly rinsed three times with distilled water to eliminate the unbound dye, and the samples were viewed under an inverted microscope to see the oil droplets. CC1 cells were cultured in 100% isopropanol to elute the Oil Red O reagent and quantify the staining. Finally, the optical density was measured at 510 nm using a microplate reader (Varioskan Flash, Thermo Scientific, Waltham, MA, USA).^22^

Lipid accumulation was quantified using Nile red fluorescent staining followed by flow cytometry (FACS Melody BD). CC1 hepatocytes were pretreated with AGN (2–105 μM) or statin (2 μM, positive control) for 2 h, then incubated with FFAs for 19 h. Cells were washed with PBS and stained with 5 μg/mL Nile red at 37 °C for 30 min in the dark. After washing with 1XPBS, fluorescence was measured with excitation at 480 and emission at 530 nm.^23^

### 2.14 The Reactive Oxygen Species (ROS) Scavenging Assay

ROS levels in CC1 cells were measured using the fluorogenic probe H_2_DCFDA. Cells (1 × 10^6^) were cultured in 24-well plates and incubated overnight, then predeveloped with AGN (2–105 μM) or statin (2 μM, positive control) for 2 h, followed by 0.75 mM FFA treatment for 19 h. After washing with PBS, cells were incubated with 10 μM H_2_DCFDA for 30 min at 37 °C in the dark. Postincubation, cells were washed, lysed with 1% Triton X-100 (170 μL), and DMSO (130 μL) was added to stop the reaction. Fluorescence was recorded in triplicate at 480 nm (excitation) and at 530 nm (emission). ROS levels were expressed relative to FFA-treated cells (set as 100%).^20^

In another series of experiments, intracellular ROS levels in CC1 cells were assessed via flow cytometry analysis using H_2_DCFDA, following the procedure outlined by ref 24 and in the method stated in Section 2.13. The fluorescence intensities of DCF were produced by intracellular ROS and then analyzed by using flow cytometry, with wavelengths at 480 and 530 nm, respectively.^24^

### 2.15 Detection of Liver Marker Enzymes

The CC1 cells were plated in a 48-well plate at 1 × 10^4^ cells/well. After 24h, the cells were processed with AGN at concentrations (2, 6, 11, 21 μM) followed by palmitate exposure. After the treatment, the cell culture supernatants were collected from different groups for 24h. The commercial kits determined the activities of SGPT and SGOT in the cell culture supernatant (Coral Clinical Systems, Uttarakhand, India).

### 2.16 Western Blot Analysis to Study the Expression of Metabolic Pathway Proteins

RIPA buffer containing phosphatase and protease inhibitors was used to lyse treated CC1 cells. A BCA assay (Thermo Scientific, USA) measured the protein content after centrifuging lysates for 30 min at 13,000 rpm. Proteins in equal quantities (20–30 μg) were separated using 10% SDS-PAGE and then put onto nitrocellulose membranes. After being blocked in 1% bovine serum albumin (BSA) for 40 min, the membranes were then treated with the appropriate primary antibody AMPK (1:1000), *p*-AMPK (1:1000), anti-HMGCR (1:1000), antiphosphorylated HMGCR (1:1000), anti-PPARα (1:1000), anti-FAS (1:1000), anti-UCP2 (1:1000), anti-SREBP1 (1:1000), and beta-actin (1:30000) and incubated overnight at 4 °C. Thereafter, membranes were washed out with TBS-T (50 mmol/L Tris–HCl, pH 7.6, mmol/L NaCl, 0.1% Tween 20) for 30 min and incubated at room temperature for 2 h with appropriate secondary antibody conjugated with HRP (1:1000 dilution). The histogram tool in Adobe Photoshop CS5 was used to determine the strength of each immunoblotting band after the blotted membrane was detected using the modified chemiluminescence method with ECL Western blotting diagnostic substrate. As an internal control, RP-conjugated-actin was utilized.^21^

### 2.17 *In silico* Analysis of Interactions Between Agnuside with HMGCR

Agnuside (AGN) interactions with HMGCR were analyzed using molecular docking and dynamic simulations. The crystal structure of a statin-based inhibitor bound 3 HMGCR was retrieved from the protein data bank (PDB) with PDB ID 3CCZ. The crystal structure was processed using the protein preparation wizard of Schrödinger Maestro. The HMGCR dimer was extracted from the tetrameric crystal structure, which is comprised of two attached inhibitors. The docking grid was generated based on both inhibitors’ binding pockets. The AGN ligand structure was prepared in Marvin Sketch and processed using the LigPrep tool of Schrödinger Maestro. The docking was performed in extra precision mode using the Glide tool. The docked complexes were extracted and subjected to molecular dynamics simulation. Furthermore, the dimeric form of inhibitor-bound HMGCR was modeled with stable conformations of AGN ligands and processed similarly.

The AGN-HMGCR complex was simulated in an orthorhombic box with a 10 Å buffer using the TIP3P water model, 0.05 M NaCl (71 Na^+^, 71 Cl^–^), and ∼ 25,322 water molecules, totalling ∼ 88,738 atoms. Energy minimization was done using the steepest descent algorithm in Schrödinger Desmond. The system was equilibrated at 300 K with NVT (120 ps, Nosé-Hoover thermostat, 1 ps relaxation) and NPT (1 bar, Martyna-Tobias-Klein barostat, 2 ps relaxation). Dynamics were integrated using the RESPA algorithm with 2 fs timesteps. A 100 ns production MD run was performed with 1 ps trajectory recording intervals.

### 2.18 ADMET Analysis

The in silico analysis of ADMET (absorption, distribution, metabolism, excretion, and toxicity) was carried out in ADMET lab 3.0 from the agnuside (AGN) structure represented in SMILES form.^25^

### 2.19 HMG-CoA Reductase Inhibition Assay

HMG-CoA reductase inhibition assay was performed using the commercially available kit (Sigma Cat No. CS1090). Initially, 20 μL of enzyme solution (0.50 mg/mL) was added to the 96-well plate, along with or without isolated agnuside (0–50 μM) in 100 μL diluted assay buffer, and incubated at 37 °C for 1 h. After the incubation, the substrate solution (HMG-CoA) and NADPH were added to the reaction mixture at volumes of 20 and 10 μL, respectively. Pravastatin (0–50 μM) is used as an enzyme inhibitor as a positive control. The absorption difference was immediately measured in the plate reader at 340 nm. Enzyme inhibition was calculated as the difference in enzyme activity per unit time between the untreated and treated Agnuside and the untreated and treated pravastatin groups. IC_50_ was calculated by interpolating the concentration vs % inhibition curve in GraphPad Prism version 5, setting Force 0 (X = 0; Y = 0).

### 2.20 Statistical Analysis

Statistical analysis was conducted using an unpaired Student’s *t*-test for comparisons between two groups, and one-way ANOVA followed by the Student–Newman–Keuls post hoc test for comparisons among multiple groups. Data are presented as mean ± SEM. Statistical significance was defined as *p* < 0.05, *p* < 0.01, and *p* < 0.001.

## 3 Results

### 3.1 The CRUDE did not Show Toxicity in CC1 Cells

The Alamar blue reduction assay investigated the effect of CRUDE on the viability of CC1 cells. The growth profile observed in the presence of different concentrations of CRUDE until 100 μg/mL (Figure 1A) was similar to that of the control. Therefore, under the conditions of our studies, the treatment of CC1 cells with CRUDE had no detectable cell toxicity.

**Figure 1:**
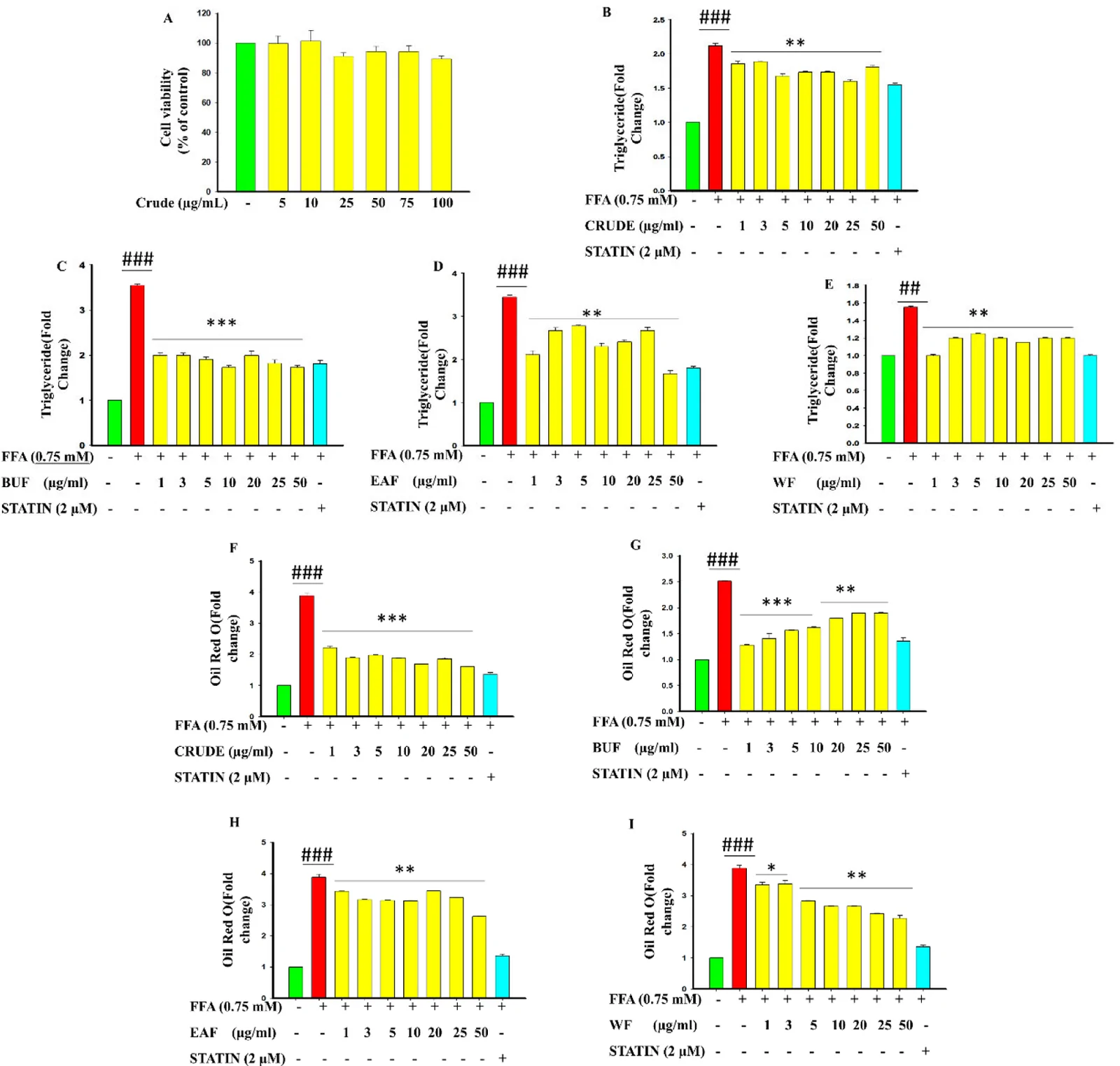
(A) *In vitro* cytotoxicity by Alamar blue assay of different concentrations of Crude (5–100 μg/mL) and Effect of intracellular triglyceride level in (B) Crude (C) BUF, (D) EAF (E) WF fractions, Also Effect of Oil Red O staining in (F) Crude (G) BUF (H) EAF (I) WF on lipid accumulation in CC1 hepatocytes exposed to high palmitate (PA). Data are expressed as mean ± SE (*n* = 3). “#” denotes the significant difference from control group (#*p* < 0.05) and “*” denotes the significant difference from PA-treated group (*p < 0.05).

### 3.2 Bioactivity Study of CRUDE and Fractions

The findings indicated a notable increase in triglyceride levels in cultured CC1 cells following exposure to palmitate. Treatment with CRUDE extract (Figure 1B) significantly decreased triglyceride levels (*p* < 0.01) in cells treated with palmitate. Also, it reduced the triglyceride levels with the treatment of BUF with *p* < 0.001­(Figure 1C) at the concentration 1–50 μg/mL. However, EAF (*p* < 0.01) (Figure 1D) and WF (*p* < 0.01) (Figure 1E) did not decrease the cellular triglyceride level compared to CRUDE and BUF.

Moreover, the results were corroborated by the lipid droplet staining method using Oil Red O, where CRUDE showed a significant reduction in the fat accumulation with a significance *p* < 0.001­(Figure 1F) compared to the palmitate-treated group and its BUF reduced lipid droplets with the significance of *p* < 0.001­(Figure 1G) at lower doses (1–10 μg/mL) compared to EAF (*p* < 0.01) (Figure 1H) and WF (Figure 1I). These studies suggest that BUF, the active fraction of CRUDE, prevents PA-induced intracellular lipid accumulation in hepatocytes.

### 3.3 Spectral Analysis of Compound 1

Three compounds were isolated from the active fraction of plant VN, of which compound 1 was the most bioactive. Compound 1 showed a deprotonated molecular ion peak at *m*/*z* 465.13989 [M-H]^−^ in the mass spectra, which refers to the molecular formula C_22_H_26_O_11_ (exact mass = 466.1475g/mol) (Figure 2A), its elemental composition is C 56.65%, H 5.62%, O 37.73%. The IR spectrum shows a broad band at 3401 cm^–1^ due to the presence of hydroxyl group. A band at 2886 cm^–1^ is due to C–H stretching. The band at 1704 cm^–1^ indicates the carbonyl group in the molecule. The band at 1511 cm^–1^ is due to aromaticity in the molecule and band at 1271 cm^–1^ can be assigned to a stretching at C–O–C (Figure 2B). Mass spectrometric analysis of vitexilactone recorded in positive ionization mode [M + H]^+^ is also shown in Figure S1.

**Figure 2:**
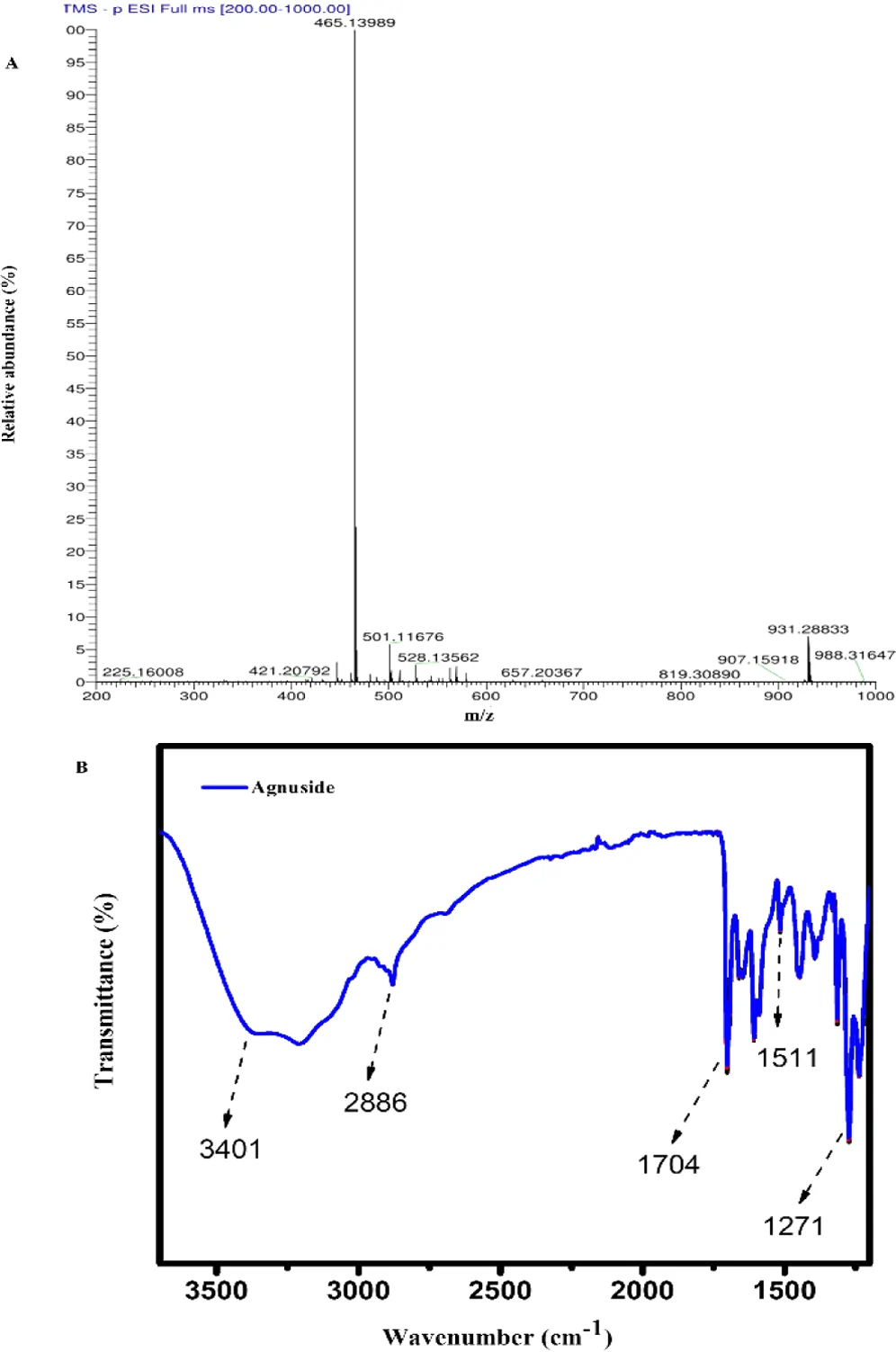
(A) Mass spectrometric analysis of Agnuside. (B) FT-IR spectra of isolated compound Agnuside.

In NMR spectrum, ^1^H ΝMR (400 MHz, DMSO): δ = 3.38 (1H, m), 3.85 (1H, t, J = 8), 5.17 (S), 5.33 (m), 5.90 (dd, 1H), 7.17 (1H, J = 8), 6.62 (1H), 5.67 (2H, m), 8.67 (2H, d, J = 8), 7.63 (2H, d, J = 8) (Figure 3A). δ = ^13^C NMR (DMSO): δ = 97.06, 141.62, 105.96, 46.09, 81.88, 132.82, 141.62, 48.01, 62.41, 97.06, 74.63, 77.92, 71.33, 78.46, 62.41, 120.63, 132.82, 116.89, 164.47, 166.52 (Figure 3B). This compound was identified as agnuside by comparison with spectroscopic data.^26^ Further, compound 2 and compound 3 were also identified to be 5-hydroxy-3,6,7,3′,4′-pentamethoxyflavone (compound 2) and Vitexilactone (compound 3) from the comparison of their spectral data with reported literature.^27^

**Figure 3:**
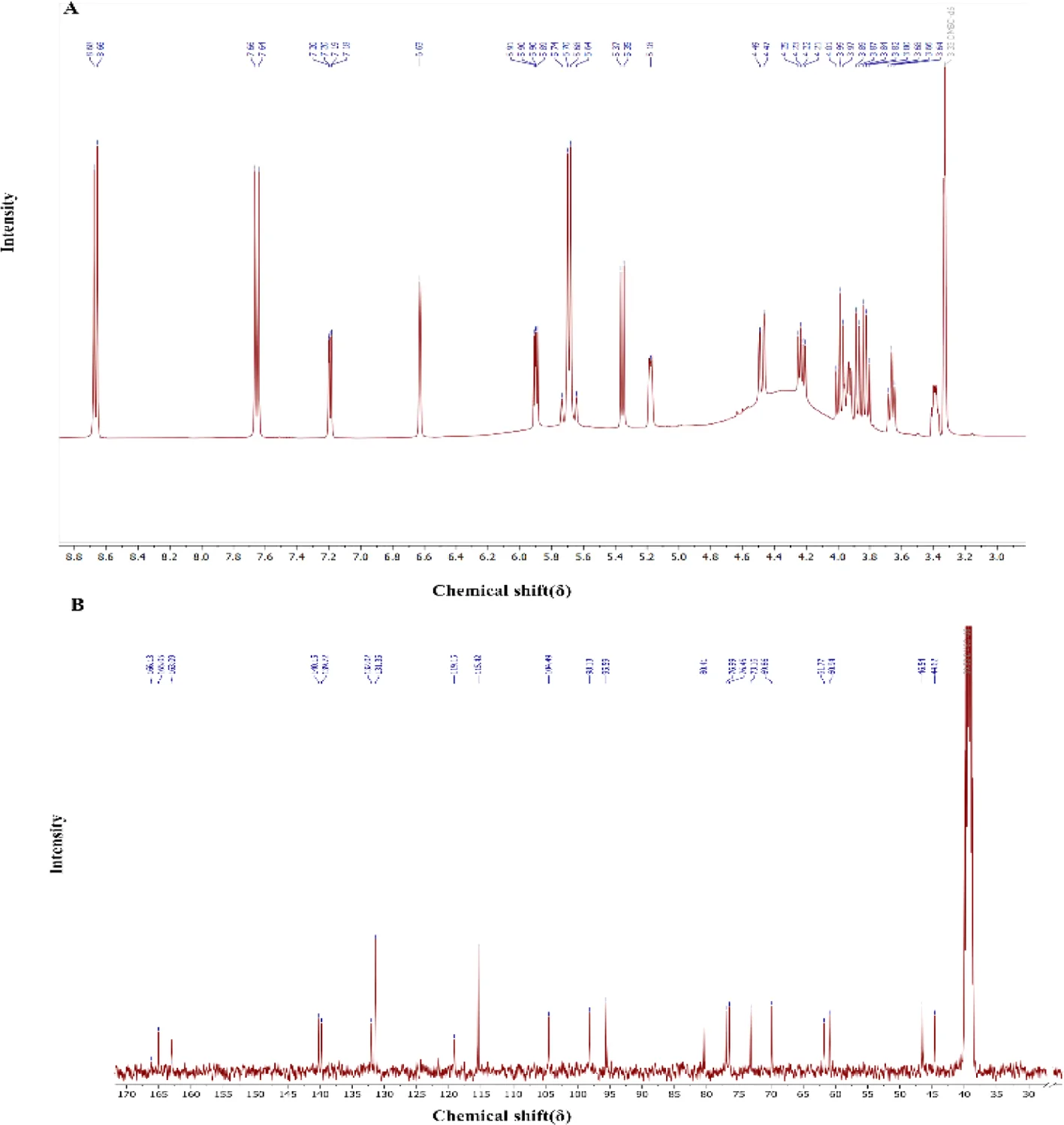
(A) The ^1^H NMR spectra of Agnuside. (B) The ^13^C NMR spectra of Agnuside.

### 3.4 Quantification of Agnuside (AGN) by HPLC-DAD

Agnuside (compound 1) was identified as the most abundant compound in the CRUDE and its BUF, as evidenced by the HPLC spectrum (Figure 4A–C). The standard AGN calibration curve, depicted in Figure 4E, demonstrated linearity (*r* ^2^ = 0.9975) within the 100 μg/mL concentration range to 900 μg/mL. Agnuside (AGN) concentration was 66.67 μg/mg in the crude extract and 680 μg/mg in the *n*-butanol fraction. The isolated Agnuside was determined to be 99.5% pure (Table 1).

**Figure 4:**
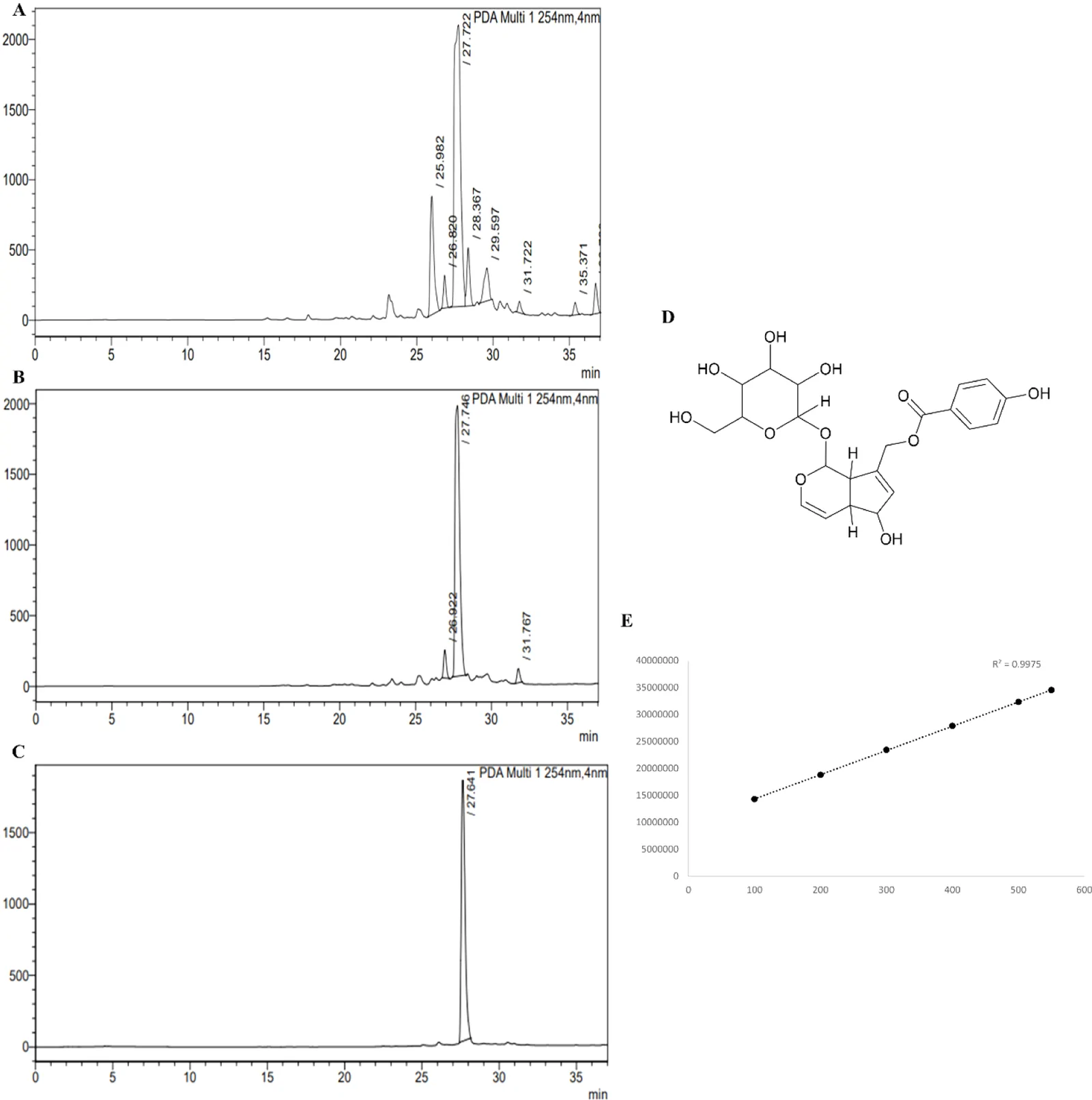
HPLC DAD quantification of AGN. HPLC chromatograms of (A) Crude, (B) BUF, (C) Isolated compound AGN. (D) Chemical structure of Agnuside (E) calibration curve was obtained using HPLC – DAD.

**Table 1:** Concentration of Agnuside in Crude, BUF and the Isolated Pure Compound, Each Value is Represented as Mean ± SD, *n* = 3

sample	Agnuside (μg/mg)
VN -crude extract	66.67 ± 2.11
VN -*n*-butanol fraction	680.5 ± 3.01
Agnuside	995.23 ± 2.08

### 3.5 Assessment of Cell Viability of Active Isolated Compound

The Alamar blue reduction assay investigated the effect of the most active compound, Agnuside (AGN), on the viability of CC1 cells. The growth profile observed in the presence of different concentrations of AGN (10 to 215 μM) (Figure 5A) was similar to that of the control cells, indicating under the conditions of our studies, the treatment of CC1 cells with AGN had no detectable cell toxicity.

**Figure 5:**
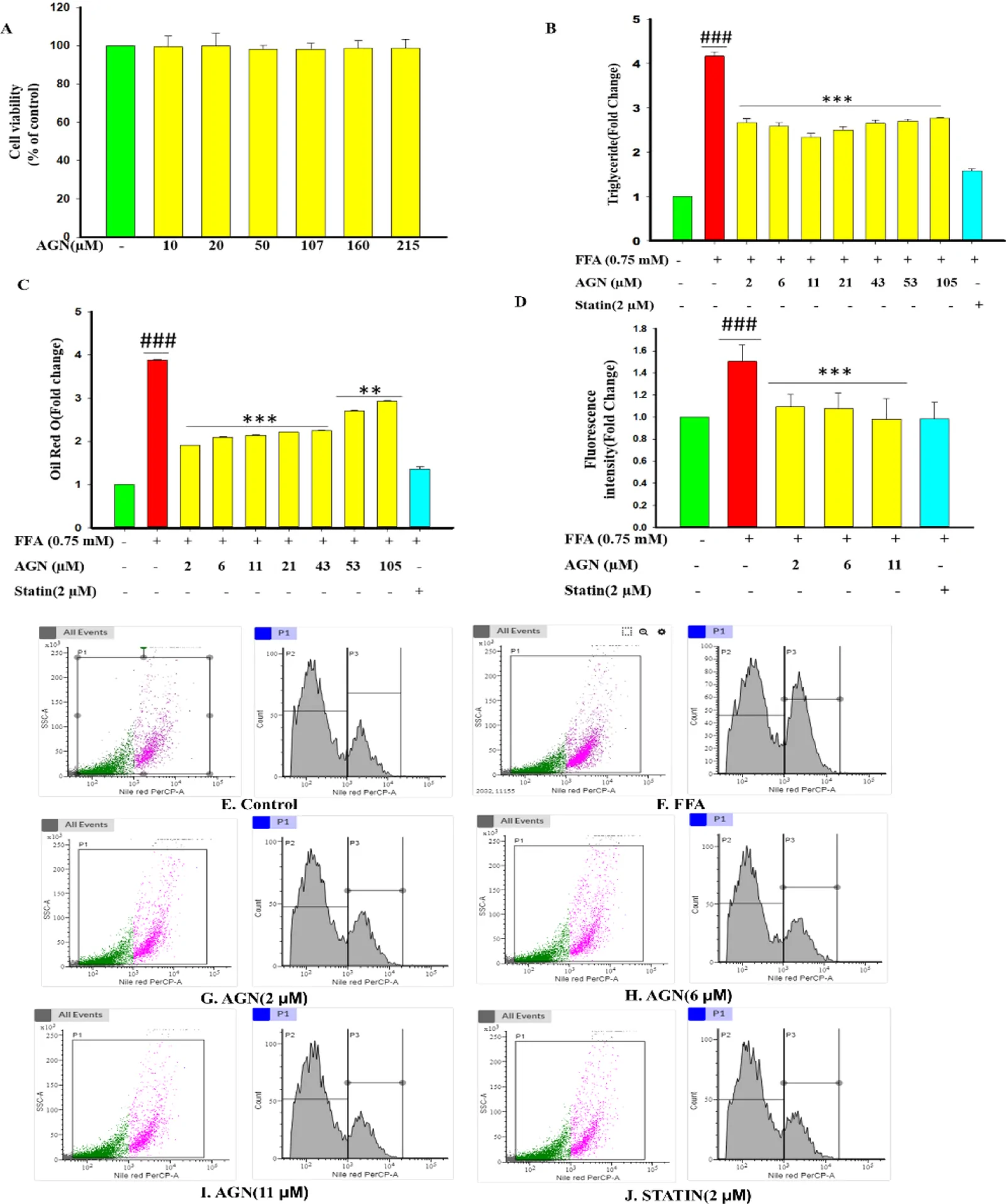
(A) *In vitro* cytotoxicity assay of different concentrations of AGN (10–215 μM) on CC1 cells (B) AGN treatment decreased Triglyceride level in CC1 cells (C)­Effect of Oil Red O on AGN markedly reduced intracellular lipid accumulation induced by palmitic acid (PA), highlighting its potential lipid-lowering effect. Flow cytometry analysis of Nile Red staining, a probe for intracellular lipid staining (D) Graphical representation flow cytometry analysis data.Flow cytometry images of (E) Control (F) FFA (G) AGN (2 μM) (H) AGN (6 μM) (I) AGN (11 μM) (J) Statin (2 μM). Data are expressed as mean ± SE (*n* = 3). “#” denotes the significant difference from control group (#*p* < 0.05) and “*” denotes the significant difference from PA-treated group (*p < 0.05).

### 3.6 The Impact of Agnuside (AGN) on Lipid Accumulation and Lipid Metabolism Signaling Pathways in Cultured Hepatocytes Exposed to Palmitate

Among the three compounds, AGN showed better-inhibited triglyceride levels at *p* < 0.001 (Figure 5B) than the FFA-treated group. The Oil Red O and triglyceride level analysis for the other two compounds has shown in the Figure S2. AGN lowered fat accumulation, or lipid droplet (Figure 5C), with a significance of *p* < 0.001 at a 2–43 μM concentration. In Oil Red O staining, pretreatment of CC1 cells with 2–105 μM of AGN significantly diminished the lipid accumulation (Figure 5C).

Further, the inhibition of fat accumulation in CC1 hepatocytes was also determined by flow cytometry analysis (Figure 5D) using Nile Red staining. Figure 5E shows untreated control CC1 cells displayed minimal lipid accumulation, while FFA-treated cells (Figure 5F) exhibited increased lipid droplets. AGN treatment significantly decreased lipid accumulation (*p* < 0.001), as observed in Figure 5G–I observed. These findings are consistent with the spectrophotometric quantification of the Oil Red O staining. It is observed that the isolated compound AGN showed better preventive intracellular lipid accumulation than the palmitate-treated group.

As shown in Figure 6A is untreated control group that shows a negligible lipid accumulation than Figure 6B, the induced group exhibited a marked accumulation of lipid droplets. However, treatment with AGN at concentrations of 2 μM and 6 μM significantly reduced lipid accumulation, as evident in Figure 6C,D, respectively, based on Oil Red O staining at 20× magnification.

**Figure 6:**
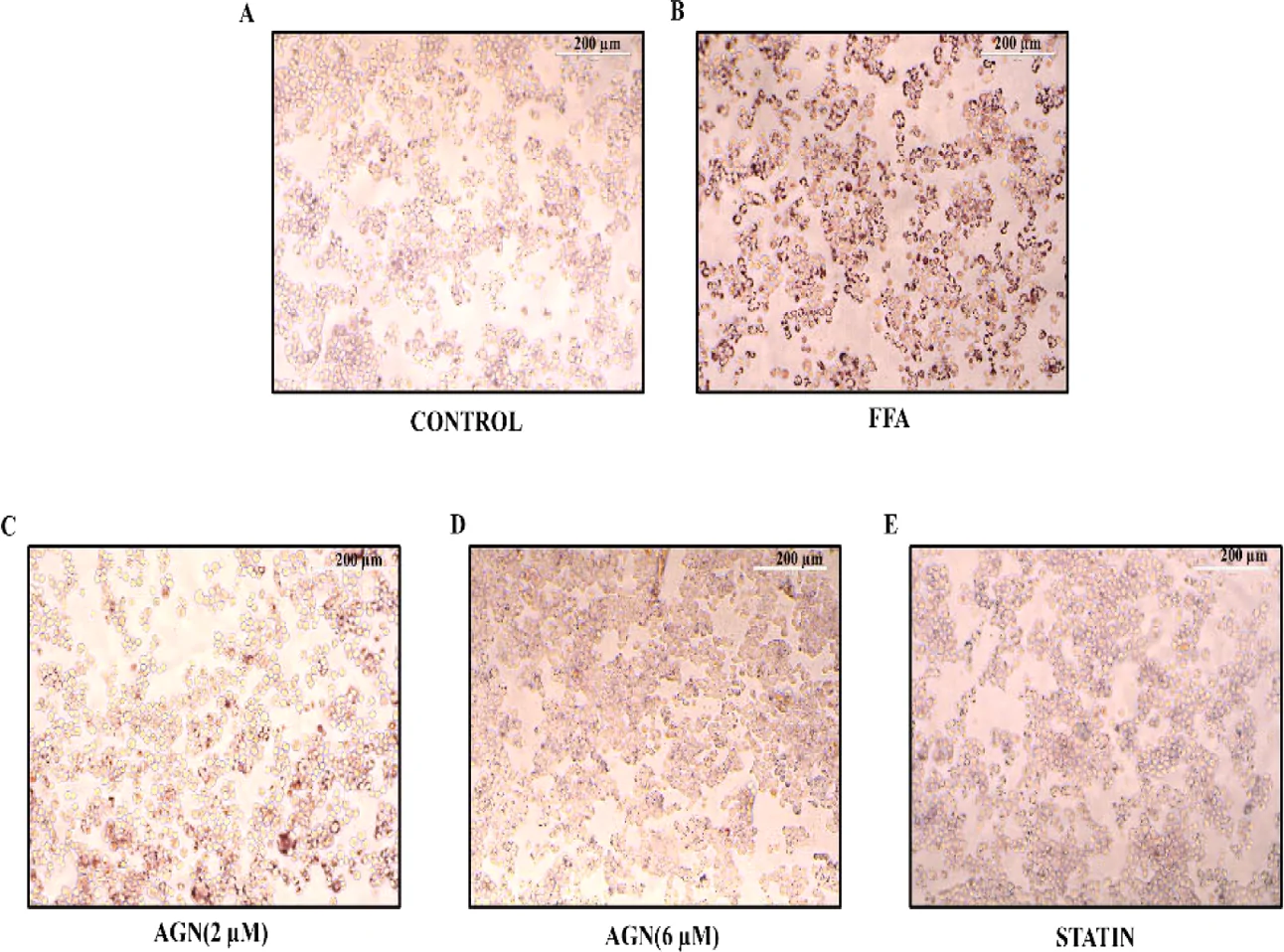
Representative photomicrographs of Oil Red O staining­(20X). (A) Control (B) FFA (C) AGN (2 μM) (D) AGN (6 μM) (E) STATIN (2 μM).

Additionally, the beneficial effects of AGN against hepatic steatosis were investigated through immunoblotting studies to elucidate the molecular mechanisms involved (Figure 7). AMP-activated protein kinase (AMPK) is the master regulator for all three critical pathways involved in lipid metabolism. The result showed that AGN-treated group with the desired doses of (2, 6, 11 μM) markedly stimulated the protein expression of phospho-AMPK in FFA-treated cells. The treatment of AGN also upregulated the expression of phospho-HMGCR, a key enzyme in mevalonate pathways and a crucial step in cholesterol biosynthesis.

**Figure 7:**
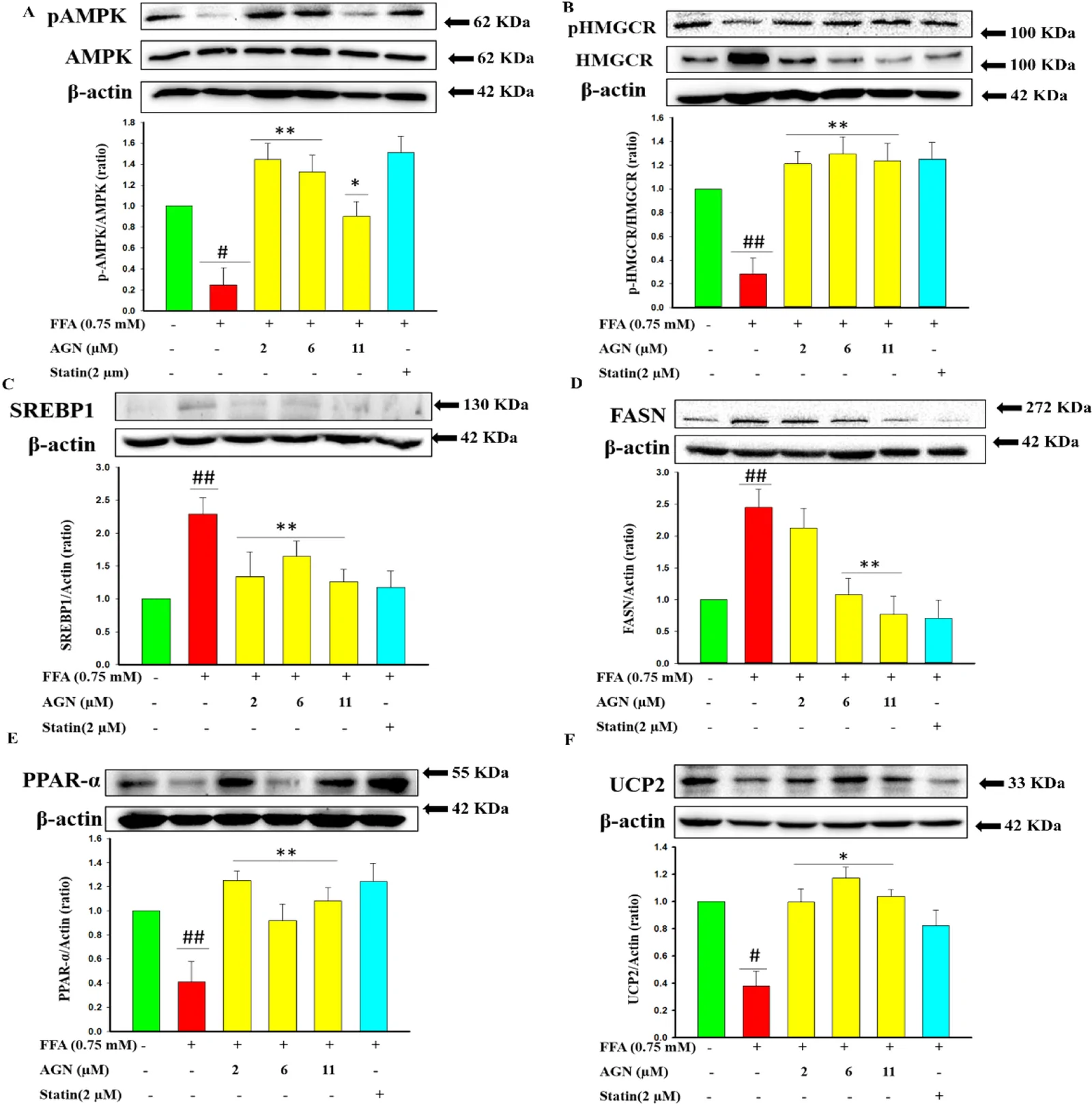
(A) Representative Western blot images of pAMPK and AMPK, level of expression pAMPK/AMPK. (B) Representative Western blot images of pHMGCR and HMGCR, level of expression pHMGCR/HMGCR. (C) Representative Western blot images of SREBP1 protein and level of phosphorylation. (D) Representative Western blot images of FASN and level of expression. (E) Representative Western blot images of PPAR-α and level of expression. (F) Representative Western blot images of UCP2 protein and level of expression. Results are mean ± SD (###) *p* < 0.001, (##) *p* < 0.01 and (#) *p* < 0.05 vs control group; (***) *p* < 0.001, (**) *p* < 0.01 and (*) *p* < 0.05 vs FFA group. *n* = 3 independent experiments for each group.

Sterol regulatory element-binding protein-1 (SREBP-1) is a key transcription factor that regulates genes in the *de novo* lipogenesis. FASN is an enzyme complex synthesizing long-chain fatty acids from acetyl CoA and malonyl CoA. AGN also lowered the protein expression for SREBP1 and FASN, which are responsible for the *de novo* lipogenesis pathway. PPAR-alpha regulates gene expression in fatty acid beta-oxidation and is a major regulator of energy homeostasis. The results indicated that PPARα and UCP2 proteins were upregulated in palmitate-induced hepatocytes. The data obtained suggest that AGN can exert a preventative effect against hepatic steatosis by modulating the AMPK/SREBP1/FASN/HMGCR/PPARα/UCP2 signaling pathway. The uncropped raw blots with marker for all the Western blot images are given in Figure S3.

### 3.7 Inhibition of FFA-Induced ROS Production by Agnuside in CC1 Cells

Spectrofluorometric analysis validated the reduction in intracellular ROS generation in CC1 cells by AGN. FFA treatment resulted in an increase in ROS production compared to control CC1 cells. A significant decrease (*p* < 0.001) in ROS levels with AGN treatment (6–21 μM) was observed in CC1 cells (Figure 8A).

**Figure 8:**
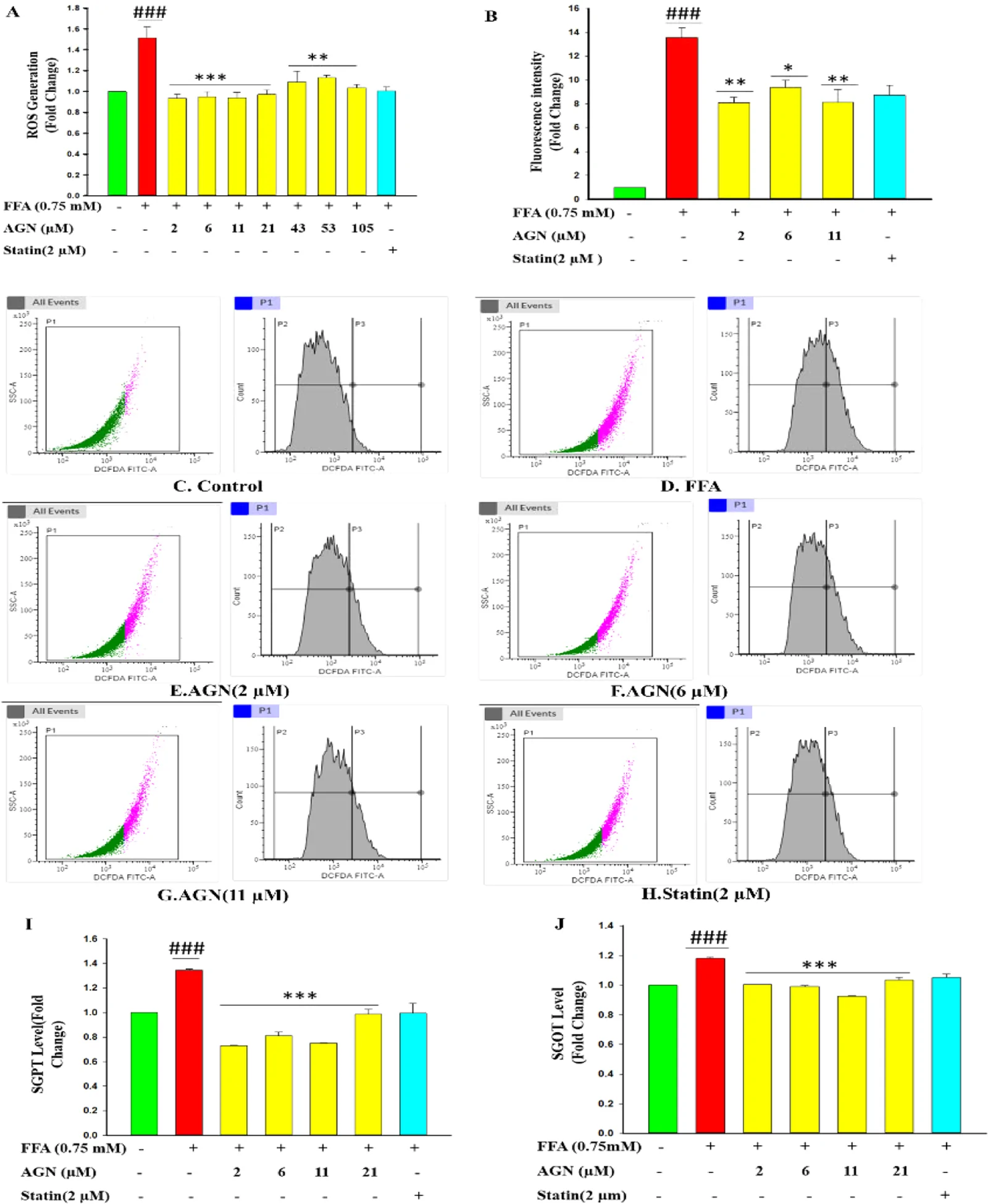
(A) AGN induced ROS generation in PA treated CC1 cells. The reduction in ROS generation in CC1 cells was also determined by flow cytometry analysis (B). Graphical representation of flow cytometry analysis data. Flow cytometry images of (C) Control, (D) FFA, (E) AGN (2 μM), (F) AGN (6 μM), (G) AGN (11 μM), (H) Statin (2 μM). The effect of AGN on PA-induced liver cell injury. AGN treatment significantly attenuated the PA-induced elevation of serum levels (I) SGPT and (J) SGOT. Data are expressed as mean ± SE (*n* = 3). “#” denotes the significant difference from control group (#*p* < 0.05) and “*” denotes the significant difference from the PA-treated group (*p < 0.05).

Flow cytometry analysis also determined the reduction in intracellular ROS generation in CC1 cells (Figure 8B). The data show a significant decrease in intracellular ROS production in control CC1 cells (Figure 8C) compared to the FFA-treated CC1 cells (Figure 8D). Nevertheless, pretreatment of CC1 cells with 6–21 μM of AGN dose-dependently diminished the ROS production (Figure 8E–G), which correlates with the fluorometric data (Figure 8B).

### 3.8 Effect of Agnuside on the Release of SGPT and SGOT in FFA-Treated CC1 Cells

The results showed that the levels of SGPT and SGOT were increased significantly in the FFA-treated CC1 cells (*p* < 0.001) (Figure 8I,J) compared with those in the control cells. A significant decrease in SGPT and SGOT levels is observed with the treatment of AGN (*p* < 0.001). The findings indicate that AGN protects liver cells against the effects of FFA.

### 3.9 Interactions with HMGCR-In Silico

The *in silico* analysis revealed that AGN can exhibit significantly stable interactions with HMGCR, similar to the interactions of the statin-based inhibitors. Two tautomeric forms of AGN, viz. AGNt1 and AGNt2 were studied for their interactions with HMGCR. The interactions of AGN tautomer containing OH group (AGNt1) were relatively promising compared to AGNt2 containing O^–^ (Figure 9A,B). AGN interacted with significant stability with dimeric forms of HMGCR as determined by RMSD analysis (Figure 9C,D). The study of RMS fluctuations of different HMGCR and ligand-interacting residues (Figure 9C).

**Figure 9:**
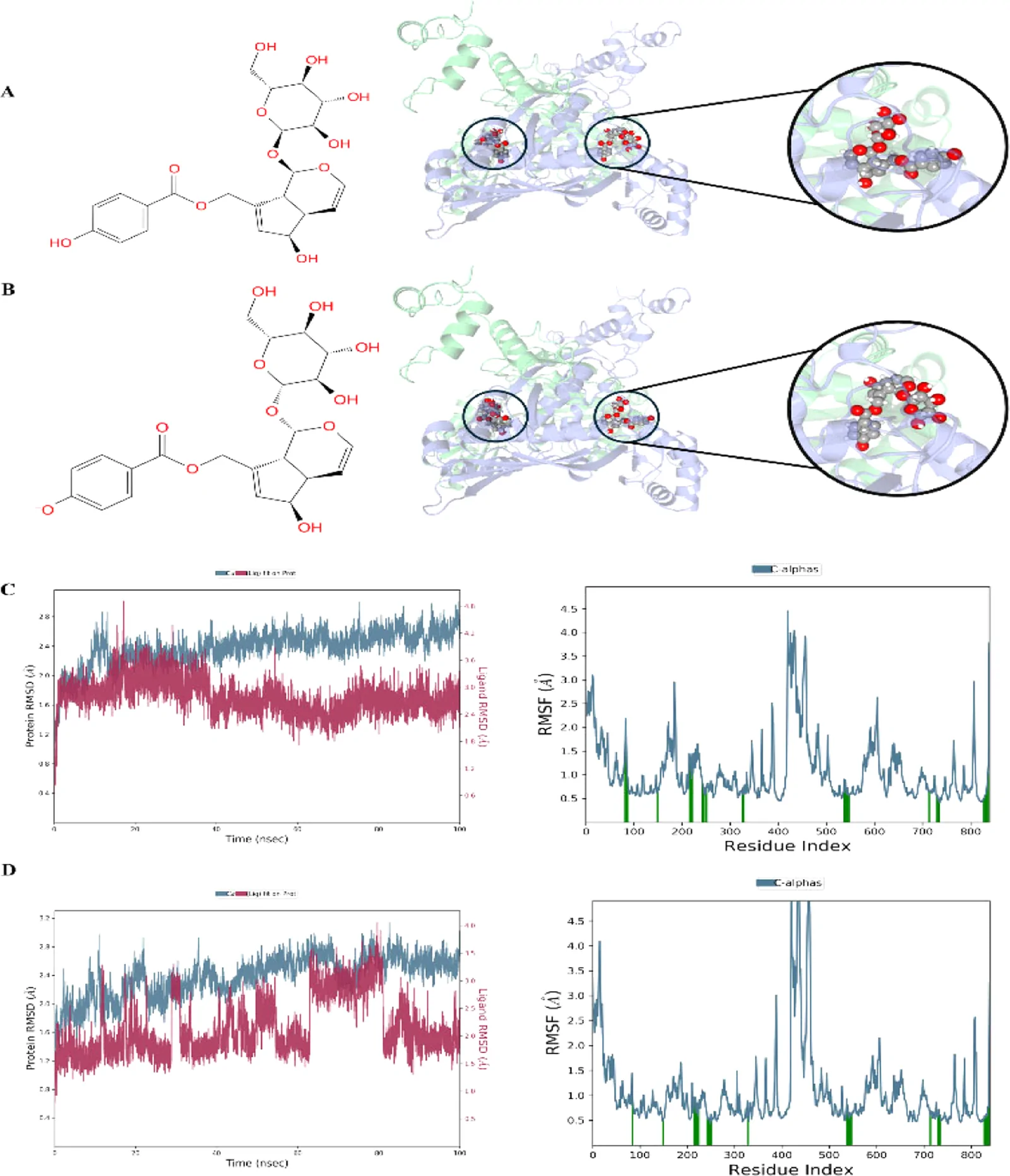
Interaction of different tautomeric forms of AGN: (A) AGNt1 and (B) AGNt2 with HMGCR dimer generated after MD simulation. RMS deviations (C) and RMS fluctuations (D) simulated the complex of HMGCR dimer and AGN tautomer - (C) AGNt1 and (D) AGNt2.

After MD simulation, the notable interactions with amino acid residues observed were with ASP 690 and ARG 590 of chain A and GLU 559, GLY 560, ARG 568, LYS 735, and HIS 752 of chain B for AGN tautomer 1 (Figure 10A,B) and ASP 690, LYS 692, SER 661, and SER 684 of chain A and MET 657, GLU 559, GLY 560, and LYS 735 of chain B for AGN tautomer 2. The binding pose of AGN inside the HMGCR dimer is symmetrical at both the binding pockets (Figure 10C–E), and docking scores were more or less similar (−8.4 to −7.0). Notably, AGN tautomer 1 was also found to interact with HMGCR via a pi–pi interaction with HIS 752. Although the AGN tautomer interacted in the same binding pocket of HMGCR, a minor variation in interacting residues was observed after MD simulation analysis. The stable dimeric and tetrameric models of HMGCR and AGN, which were derived from stable complexes after MD simulation, are shown in (Figure 10C–E), which highly resembled the previously reported crystal structures of HMGCR (PDB ID: 3CCZ) and even CoA-bound HMGCR crystal structures (PDB ID: 1DQ8).

**Figure 10:**
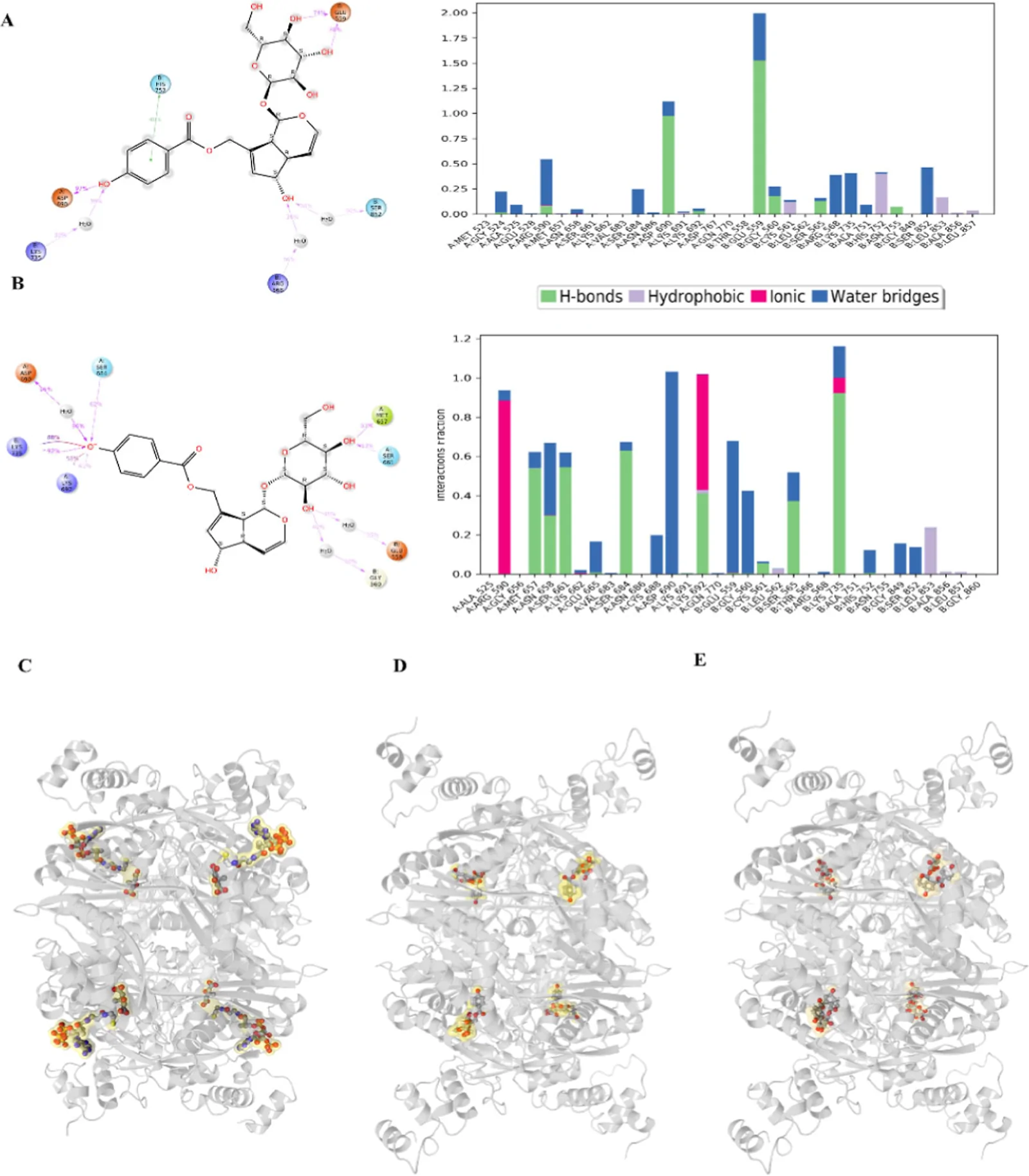
Interaction fractions of noncovalent interactions of amino acid residues in HMGCR dimer with different tautomeric forms of AGN: (A) AGNt1 and (B) AGNt2. Tetrameric crystal structure of HMGCR in complex with HMG and CoA (C) and Modeled tetrameric structures of HMGCR and AGN complexes: (D) AGNt1 and (E) AGNt2.

### 3.10 ADMET Analysis

The ADMET profile of AGN was evaluated using in silico predictions, as detailed in Table S1. AGN satisfies Lipinski’s rule of five without violations, indicating physicochemical properties generally compatible with those of drug-like small molecules. The predicted ADME parameters showed generally favorable profiles with high probability scores; however, the predicted permeability and toxicity profiles indicate potential limitations and safety liabilities. Specifically, adverse toxicity predictions were noted across several parameters with high probability scores (Table S1).

Although favorable profiles were predicted for certain specific end points, namely reproductive toxicity, mitochondrial toxicity, nephrotoxicity, and endocrine interactions (estrogen, androgen, thyroid, and aromatase binding), these nontoxic classifications were associated with low probability scores and should be interpreted with caution. Furthermore, while the molecular descriptor radar plot (Figure S4) indicates overall drug-likeness, the ADMET conclusions must be tempered in light of these predicted toxicity risks and potential permeability constraints. To complement the in silico findings with functional data, an in vitro enzyme inhibition assay against HMG-CoA reductase was conducted. AGN exhibited an IC_50_ of 14.68 ± 4.56 μM compared to 5.65 ± 1.89 μM for the reference inhibitor, pravastatin, under identical conditions (Figure S5).

## 4 Discussion

VN, a small tree common in wastelands, has been traditionally used for its medicinal properties, with leaf and bark extracts showing strong therapeutic effects. It is primarily known for its anti-inflammatory and hepatoprotective activities. AGN, an iridoid glycoside and key active compound in the Vitex genus, is found in VN and other species like *Vitex agnus-castus* and *Vitex cymosa*. Composed of aucubin and *p*-hydroxybenzoic acid, AGN has shown antiarthritic and anti-inflammatory effects by modulating immune responses. Its broad pharmacological profile makes it a promising candidate for liver disease therapy.^12,28,29^

On the basis of existing literature highlighting the therapeutic potential of various *Vitex* species and compounds derived from VN, the present study further validates the significant role of VN and its bioactive constituent, AGN, in regulating hepatic lipid metabolism, a key factor in the progression of lipid accumulation CC1 hepatocytes. In this context, bioactivity-guided screening of CRUDE and its fractions identified BUF as the most effective in reducing the intracellular lipid accumulation and cellular triglyceride levels in hepatocytes. This observation suggests that its bioactive components may help prevent lowering of the triglyceride level and lipid accumulation. Further isolation yielded three phytoconstituents with AGN identified as the key compound. Structural characterization was performed by using LC–MS and NMR techniques.

AGN, with the molecular formula C_22_H_26_O_11_, was confirmed through HRMS (Figure 2A), along with detailed ^1^H and ^13^C NMR data. Furthermore, initial *in silico* QSAR and ADMET predictions tentatively indicated a tolerable drug–likeness profile, supporting the continued evaluation of AGN against hepatic lipid dysregulation.

The experiment continued by evaluating intracellular lipid buildup, a characteristic of NAFLD, by Oil Red O and Nile Red staining. Oil Red O, a lysochrome and azo dye, exhibits unique binding affinity for neutral lipids, including triglycerides and lipoproteins. Due to its inadequate ionization and elevated lipid solubility, it proficiently stains lipids with a red hue and exhibits maximum absorbance at 518 nm. This staining technique is extensively employed to visualize and quantify hepatic lipid buildup, facilitating the assessment of NAFLD.^30^ Nile Red, a lipophilic fluorescent dye, is another widely used marker that fluoresces strongly when intercalated into intracellular lipid droplets, enabling sensitive detection and quantification of neutral lipids under fluorescence microscopy.^30^ In this study, Oil Red O and Nile Red staining revealed that treatment with BUF at concentrations of 1, 3, and 5 μg/mL significantly reduced lipid accumulation in palmitic acid (PA)-treated hepatocytes. Similarly, AGN treatment led to a marked reduction in Oil Red O staining compared to the FFA-treated control group. AGN and BUF also significantly decreased intracellular triglyceride levels (*p* < 0.001), reinforcing their role in mitigating hepatic lipid overload. Notably, this is the first report demonstrating AGN’s protective effects against hepatic steatosis, highlighting its potential as a novel therapeutic agent for steatosis in liver.

Following the reduction of cholesterol accumulation by BUF and AGN, mechanistic tests were performed to elucidate AGN’s function in hepatic lipid metabolism. Liver lipid homeostasis is essential, and disturbances such as elevated free fatty acid input, poor β-oxidation, excessive *de novo* lipogenesis, or cholesterol accumulation can lead to hepatic lipid accumulation. Of them, only β-oxidation markedly diminishes lipid surplus. SREBP1, which stimulates FASN genes, governs *de novo* lipogenesis, a crucial hepatic lipid synthesis.^31,32^ HMG-CoA reductase, the rate-limiting enzyme in converting HMG-CoA to mevalonate, is a pivotal step in total cholesterol synthesis and regulates cholesterol biosynthesis. AMPK regulates essential transcription factors to control hepatic lipid metabolism.^33^ SREBP1 enhances lipogenesis, whereas PPARα elevates fatty acid oxidation and regulates genes such as UCP2, facilitating lipid catabolism. AMPK modulates hepatic lipid synthesis and degradation via this regulatory axis.^8,34^

In our study, immunoblotting analysis revealed that AGN significantly downregulated the expression of SREBP1 and FASN, thereby inhibiting *de novo* lipogenesis. In contrast, AGN upregulated the expression of UCP2, suggesting enhanced fatty acid oxidation via PPARα signaling and downregulated HMGCR. These immunoblotting analyses revealed targeted effects of AGN on hepatic lipogenic and fatty acid oxidation proteins involved in lipid metabolism, offering insights into its therapeutic potential for managing liver fat accumulation. MASLD is increasingly recognized as a systemic metabolic disorder closely associated with obesity, insulin resistance, dyslipidemia, and an elevated risk of cardiovascular disease (CVD), the leading cause of mortality in affected patients.^35,36^ Activation of AMP-activated protein kinase (AMPK) and inhibition of 3-hydroxy-3-methylglutaryl-coenzyme A reductase (HMGCR) regulate hepatic lipid and cholesterol metabolism and may also improve systemic metabolic homeostasis. Therefore, natural products targeting these pathways have emerged as promising multitarget therapeutic agents. In this study, agnuside-mediated AMPK activation and HMGCR suppression suggest potential benefits beyond hepatic lipid accumulation by modulating shared mechanisms involved in MASLD and cardiometabolic disease.^37^

Moreover, the present study revealed that apart from regulating HMGCR expression, AGN was also found to interact significantly with HMGCR in the binding site, which interacts with HMG-CoA (Figure 10A,B). This result may also exhibit inhibitory responses toward HMGCR activity. Similar interactions were reported earlier with statin-based inhibitors, and the nature of the interactions corroborates with the interactions present in silico analysis.^38^ For instance, statin-based inhibitors often interact with ARG 590, GLU 559, SER 684, and LYS 735 of HMGCR; in our case, AGN exhibited strong interaction with these residues by hydrogen bonding (Figure 9A,B). These data suggest that AGN can potentially act as an inhibitor of HMGCR as well as affect multiple metabolic pathways, preventing liver *de novo* lipogenesis.^38^

Mitochondria, the cell’s powerhouse, are crucial for energy production and lipid metabolism. Their dysfunction disrupts lipid homeostasis and contributes to NAFLD.^39^ In hyperlipidaemia, excess FFAs accumulate in the liver. When unmetabolized, these FFAs cause hepatic steatosis and generate lipid intermediates that promote ROS production.^40^ Lipid overload and mitochondrial impairment form a self-perpetuating cycle, each worsening the other. Restoring mitochondrial function is thus a promising strategy to reduce lipid accumulation and oxidative stress, consistent with studies showing antioxidant protection against high-fat diet-induced NAFLD.^41–43^ Agnuside attenuates hepatocellular lipid accumulation by suppressing oxidative stress generated during ethanol metabolism, preventing acetaldehyde-induced mitochondrial damage, and suppressing NF-κB-dependent inflammatory signaling. Moreover, it enhances endogenous antioxidant defenses, especially glutathione-related pathways, restoring redox homeostasis and alleviating oxidative stress that causes alcohol-induced steatosis. Experimental studies have also revealed the ability of agnuside to improve hepatocellular membrane integrity, reduce lipid peroxidation and inhibit Kupffer cell activation, indicating a multifaceted hepatoprotective effect. These mechanisms have been mostly reported in the setting of alcohol-associated liver disease (AFLD), however they substantially overlap with the pathological processes involved in nonalcoholic fatty liver disease (NAFLD) including oxidative stress, inflammation, mitochondrial dysfunction and hepatic lipid accumulation. Thus, the hepatoprotective effects of agnuside are consistent with the traditional view that NAFLD and AFLD share many molecular and pathological mechanisms despite their different etiologies.^44^ Our study demonstrated a significant reduction in ROS levels (*p* < 0.001) in CC1 hepatocytes treated with AGN. These results suggest that AGN effectively attenuates oxidative stress and improves mitochondrial dysfunction. Although AGN is known for a broad spectrum of pharmacological activities, our findings provide preliminary evidence of its potential to reduce hepatocellular lipid accumulation *in vitro*.

Furthermore, hepatic lipid accumulation is frequently associated with the release of intracellular enzymes such as SGPT and SGOT, critical liver damage biomarkers. Our study demonstrated that AGN administration markedly decreased the SGPT and SGOT levels, suggesting its hepatoprotective efficacy. These data demonstrate that AGN mitigates oxidative damage and safeguards liver function in FFA-induced CC1 cells, supporting its therapeutic potential for the management of liver fat deposition.

## 5 Conclusion

The current study demonstrated that AGN, an active constituent among the three phytoconstituents isolated from VN, reduces lipid accumulation in *in vitro* accompanied by modulations in the AMPK/SREBP1/FASN/HMGCR/PPAR-α/UCP2 signaling axis. Also, AGN shows moderate inhibitory activity against HMGCR in *in silico* analysis. AGN was identified by chemical profiling as a substantial bioactive component that contributes to its protective activity against palmitate-induced fat deposition. These results point to the potential of *V. negundo*, a nutrient-rich dietary food, as a cutting-edge therapeutic approach for treating disrupted lipid homeostasis. Nevertheless, further *in vivo* studies and translational clinical trials involving target knockdowns or functional rescue models are required to definitively establish direct causality and therapeutic efficacy.

## Supplementary Material



## Notes

Instrumental setup and mass spectral data analysis; Mass spectrometric analysis of vitexilactone recorded in positive ionization mode [M + H]^+^; Triglyceride level of (A) PMF (B) VL and Oil Red O quantification of (A) PMF (B) VL; Full-length blots for Western blotting studies of (A) AMPK (B) pAMPK (C) HMGCR (D) pHMGCR (E) SREBP1C (F) FASN (G) PPARα (H) UCP2; RADAR plot showing drug likeliness of AGN, represented by values of different chemical descriptors and their acceptable upper and lower limit values; Predicted ADMET classification parameters for agnuside with probabilities of their occurrence; HMG-CoA Reductase Inhibition Assay (PDF)

## Abbreviations

NAFLD: Non Alcoholic Fatty Liver Disease
Nonalcoholic Steato-Hepatitis: NASH
NAFL: Nonalcoholic Fatty Liver
FFA: Free fatty acid
ROS: Reactive Oxygen Species
DNL: *de novo* Lipogenesis
SREBP1c: Sterol Regulatory Element-Binding Protein 1c
AMPK: AMP-activated protein kinase
FASN: Fatty Acid Synthase
HMGCoA: 3-hydroxy-3-methylglutaryl-Coenzyme A
HMGCR: 3-hydroxy-3-methylglutaryl-Coenzyme A reductase
UCP2: Uncoupling protein 2
PPARα: Peroxisome proliferator-activated receptor alpha
VN: *Vitex negundo*
Crude: *Vitex negundo* methanolic extract
BUF: *Vitex negundo n*-butanol fraction
EAF: *Vitex negundo* Ethyl Acetate fraction
WF: *Vitex negundo* Water fraction
NMR: nuclear magnetic resonance
HPLC: High-Performance Liquid Chromatography
SGPT: Serum Glutamic Pyruvic Transaminase
SGOT: Serum glutamic-oxaloacetic transaminase.
