# Supplementary material for: Agnuside from Vitex negundo L. Attenuates Hepatocellular Lipid Accumulation: Association with the AMPK/SREBP1/FASN/HMGCR/PPARα/UCP2 Signaling Network
Source: ACS Omega. 2026 Oct 28;11(44):68632–47. doi: 10.1021/acsomega.6c06583 (PMC13757082; doi:10.1021/acsomega.6c06583)

Agnuside from *Vitex negundo* L. Attenuates Hepatocellular Lipid Accumulation: Association with the AMPK / SREBP1 / FASN / HMGCR / PPARα / UCP2 Signaling Network

Abhipsha Saikia[a,b], Semim Akhtar Ahmed[a], Kangkon Saikia[a], Pranamika Sarma[a], Devi Basumatary[a], Ashis K. Mukherjee[a,b,c], Jagat Chandra Borah[a,b,d*], Asis Bala[a,b*],

Sl. No	Description	Pages
1.	MS. Instrumental setup and mass spectral data analysis.	S2-S3
2.	Supplementary Fig 1: Mass spectrometric analysis of vitexilactone recorded in positive ionization mode $[M+H]^+$.	S4
3.	Supplementary Fig 2: Triglyceride level of (A) PMF (B) VL.Oil Red O quantification of (A) PMF (B) VL.	S5
4.	Supplementary Fig 3: Full-length blots for Western blotting studies of (A) AMPK (B) pAMPK (C) HMGCR (D) pHMGCR (E) SREBP1C (F) FASN (G) PPARα (H) UCP2	S6-S8
5.	Supplementary Fig 4: RADAR plot showing drug likeliness of AGN, represented by values of different chemical descriptors and their acceptable upper and lower limit values.	S9
6.	Supplementary Table S1. Predicted ADMET classification parameters for agnuside with probabilities of their occurrence.	S10-S22
7.	Supplementary Fig 05. HMG-CoA Reductase Inhibition Assay.	S23

MS: Instrumental setup and mass spectral data analysis:

MS1.Instrument:

An Orbitrap Mass Spectrometer (Thermo Scientific Exactive Plus Orbitrap) with ion sources ESI from Thermo Scientific was used to obtain the mass spectra. Nuclear magnetic resonance spectroscopy was recorded in 400 MHz FT–NMR instruments (Brukar Avance II). Chemical shifts (δ) are given in parts per million and coupling constant (*J*) in hertz (Hz).

MS2. Structure elucidation and identification of the isolated compound

The isolated compound was identified using spectroscopic techniques, specifically NMR and mass spectrometry. Proton (^1H) and carbon-13 (^{13}C) NMR spectra were recorded on a 400 MHz FT-NMR instrument (Bruker Avance II). The mass spectrum of the isolated compound was recorded using the Thermo Exactive plus Orbitrap Mass Spectrometer (Thermo Scientific), which is equipped with an electrospray ionization (ESI) interface. Mass was recorded by directly injecting 10µl of the sample into a mass spectrometer. The recorded mass spectrum was further analyzed using Xcalibur software.

Supplementary data S1: Spectral data of the isolated compounds 2 And 3

5-hydroxy-3,6,7,3',4'-pentamethoxyflavone: Compound 2, identified as 5-hydroxy-3,6,7,3’,4’-pentamethoxyflavone was isolated as yellowish color powder. The mass spectral data showed $[M+H]^+$ peak at 389.1138, corresponding to the molecular formula $C_{20}H_{20}O_8$(388.1158 g/mol). The NMR spectrum shows a signal at 7.076 ppm (1 H, doublet, J = 9 Hz) and another at 7.856 ppm (2 H, multiplet), which suggests a 3',4'-disubstituted B-ring. A singlet at 6.5 ppm (1 H) is assigned to an uncoupled proton in the ring. A low-field one-proton singlet at 12.576 ppm, which disappears upon D_2O exchange, indicates a 5-hydroxyl group. A comparison of the spectral data with

reported literature suggests that compound 2 to be 5-hydroxy-3,6,7,3',4'-pentamethoxyflavone is given below:

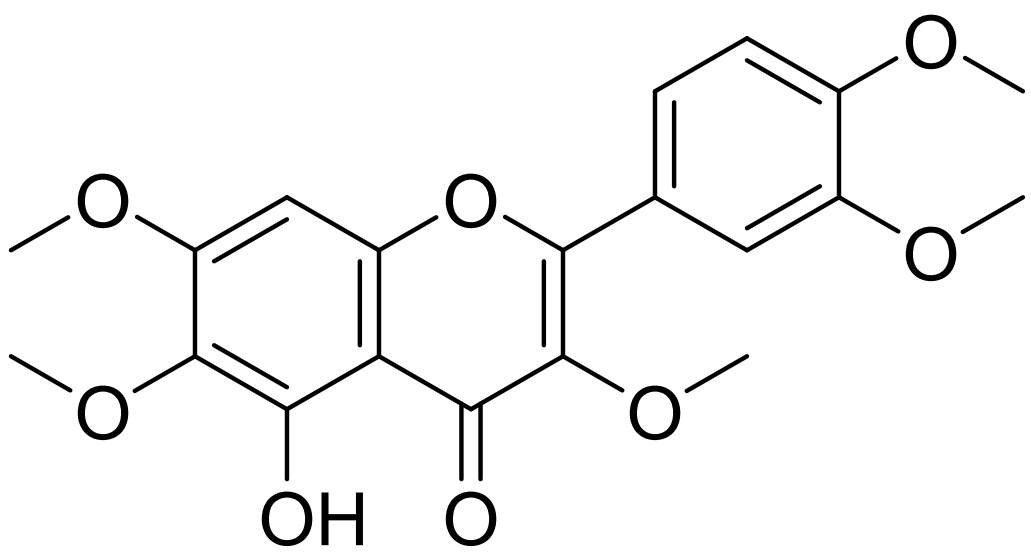

Vitexilactone: Compound 3 was isolated as an odorless white powder and identified as vitexilactone.Mass spectral analysis exhibited a protonated molecular ion peak at m/z 379.24788 $[M+H]^+$ (Calculated for $C_{22}H_{35}O_5^+$, 379.24790) consistent with the molecular formula $C_{22}H_{34}O_5$ (Δ= - 0.05 ppm). Comparison of this spectral information with published reference data confirmed the identification of the compound as vitexilactone. In NMR spectrum (400 MHz, $CDCl_3$) ^1H NMR 1.11 (1H, m),1.37(1 H, m),1.61(1H, m), 1.76(1H, m), 1.46(1H, m), 1.51(1H,m), 1.55(1H, m), 5.39(1H, s), 1.56(2H, m), 2.11(1H,m), 1.71(1H,m), 1.91(1H,m), 2.52(2H,t, J = 8.3Hz), 5.85(1H,s), 4.76(2H,s), 0.92(3H,d, J=6.8 Hz), 1.28(3H,s), 0.98(3H,s), 1.03(3H,s), 2.06(3H,s).^{13}C NMR: 44.1,19.2, 33.6, 43.9, 47.9, 69.9, 36.5, 32.5, 77.6, 35, 32.6, 25.7, 171,115.6, 174.1, 73.6, 16.4, 18.9, 33.9, 23.9, 170.7, 23. The structure of Vitexilactone is provided below-

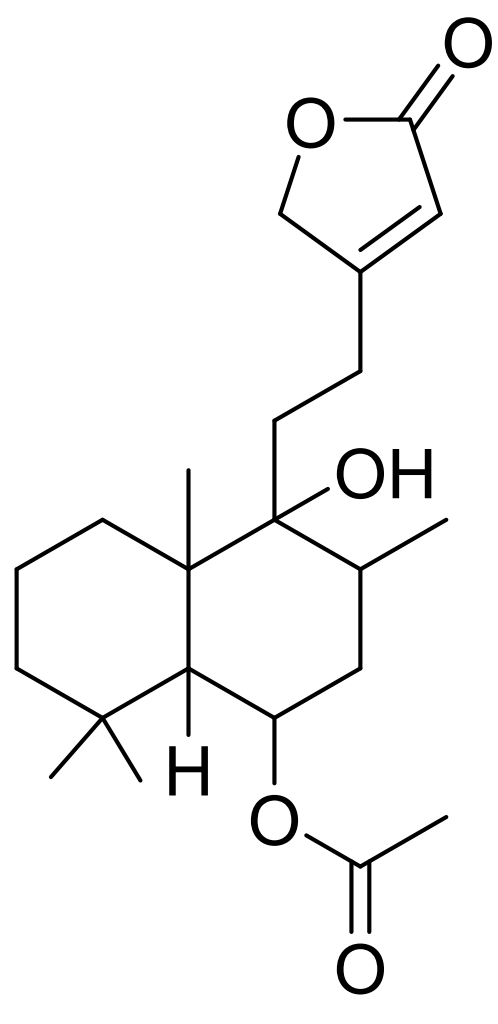

Supplementary Fig 1: Mass spectrometric analysis of vitexilactone recorded in positive ionization mode $[M+H]^+$.

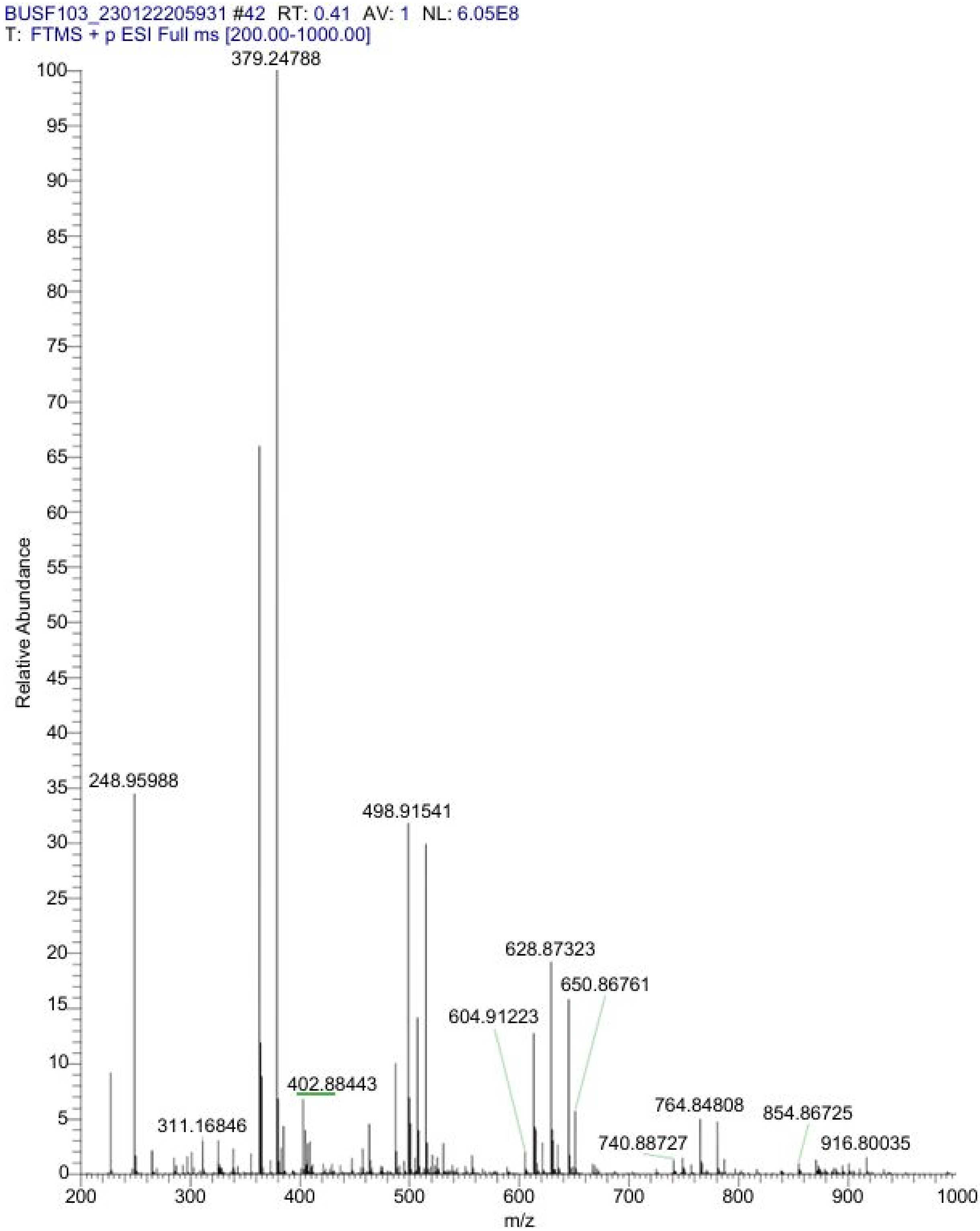

Supplementary Fig 2: Triglyceride level of **(A)** PMF(5-hydroxy-3,6,7,3',4'-pentamethoxyflavone) **(B)** VL (Vitexilactone).Oil Red O quantification **(C)**PMF **(D)** VL. Data are expressed as mean ± SE (n=3). “#” denotes the significantdifference from control group (#p < 0.05) and “*” denotes the significant difference from PA-treated group (*p < 0.05).

Compared with Agnuside, PMF and VL exhibited variable and less consistent reductions in both Oil Red O staining and intracellular triglyceride levels, indicating comparatively lower lipid-lowering efficacy.

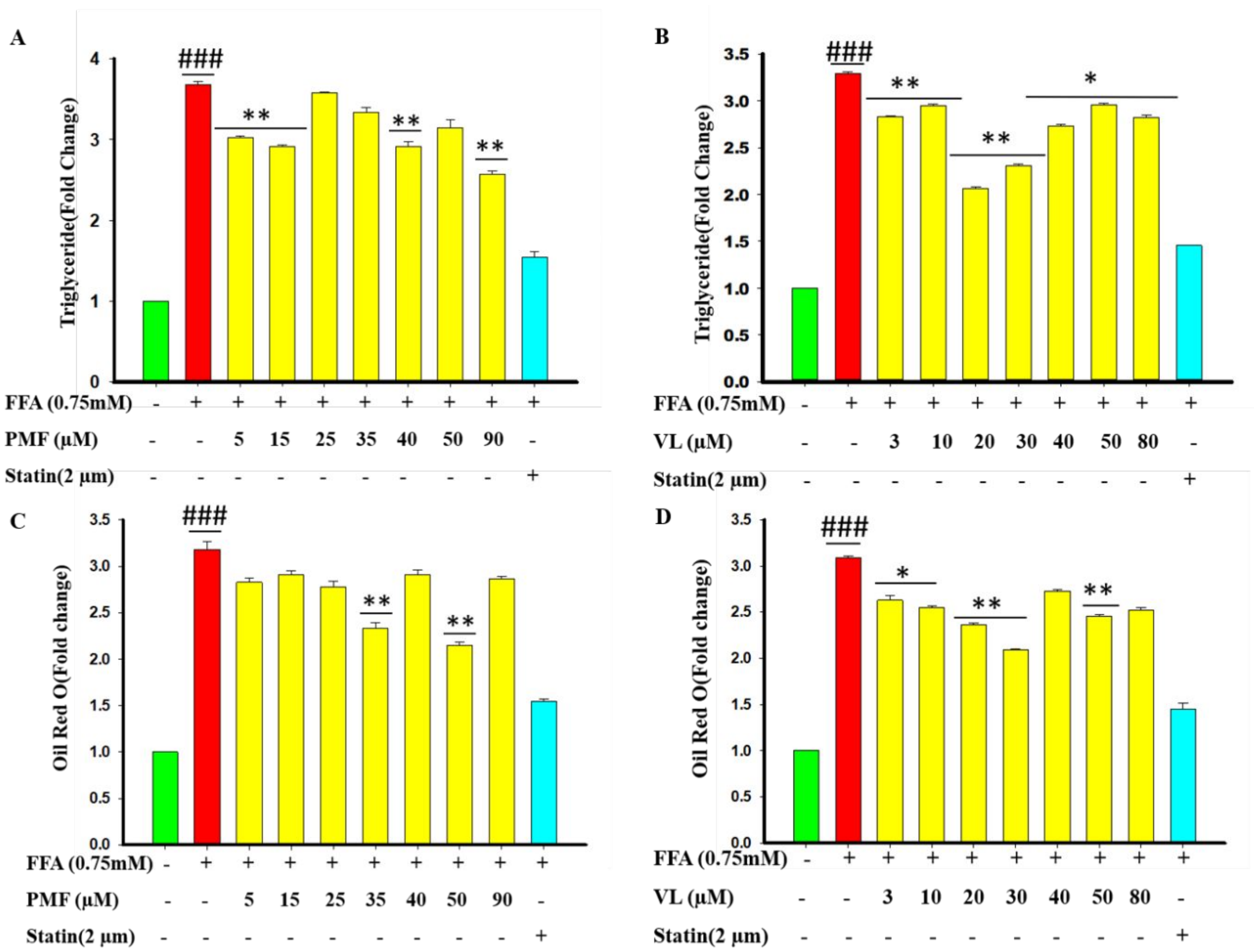

Supplementary Fig 3: Full-length blots for Western blotting studies of (A) AMPK (B) pAMPK (C) HMGCR (D) pHMGCR (E) SREBP1C (F) FASN (G) PPARα (H) UCP2

(A) AMPK

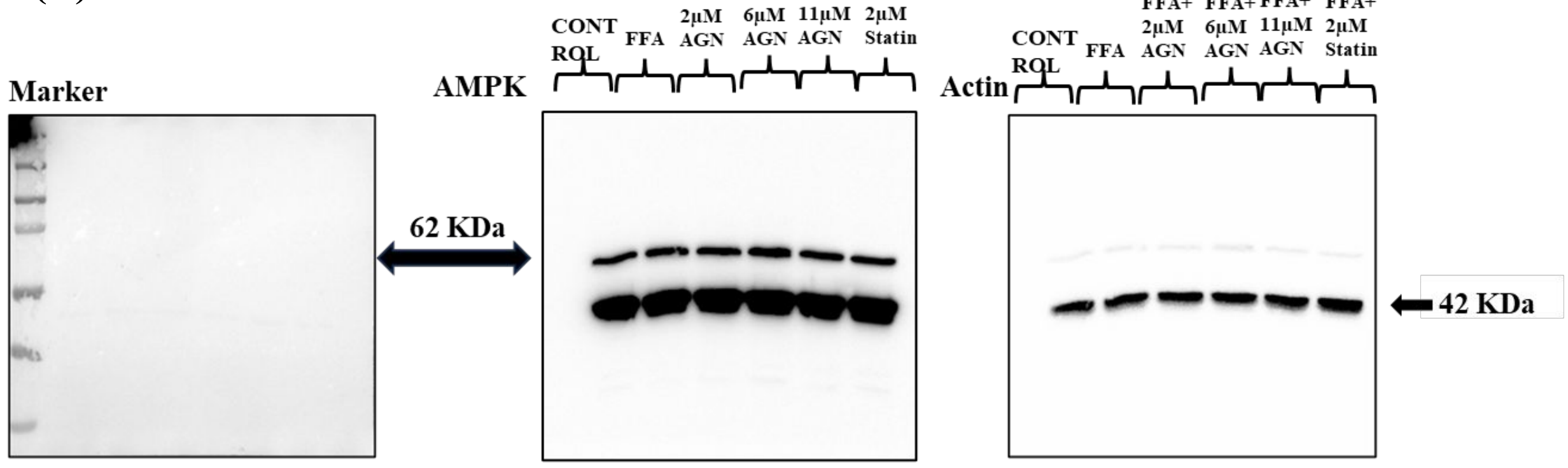

(B) pAMPK

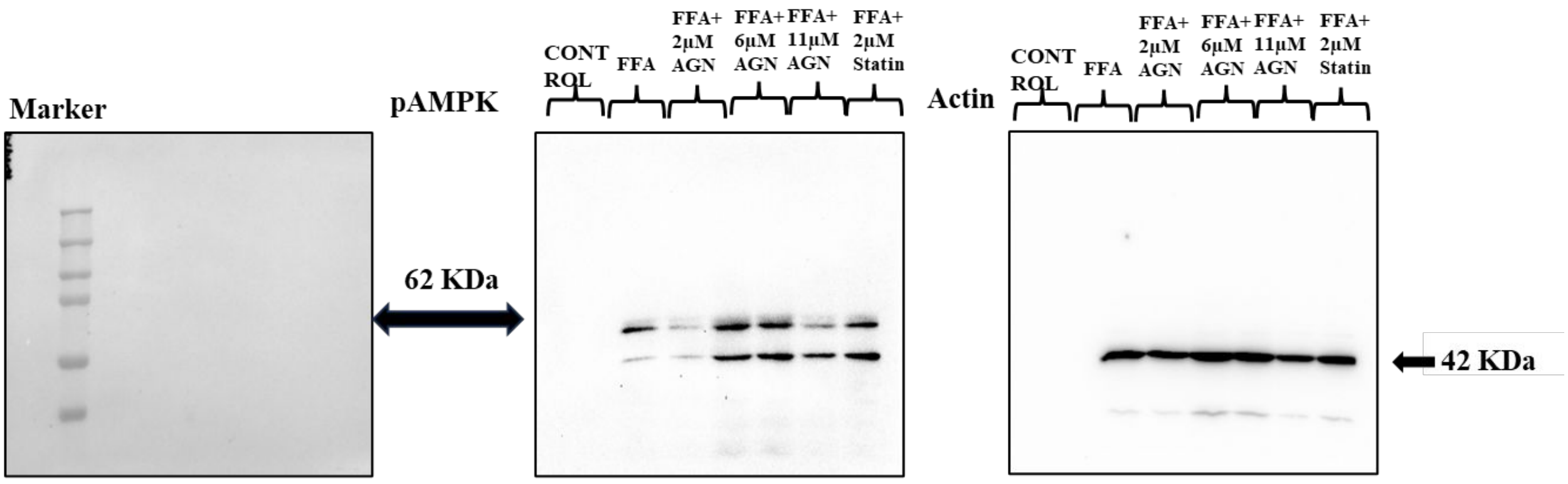

(C) HMGCR

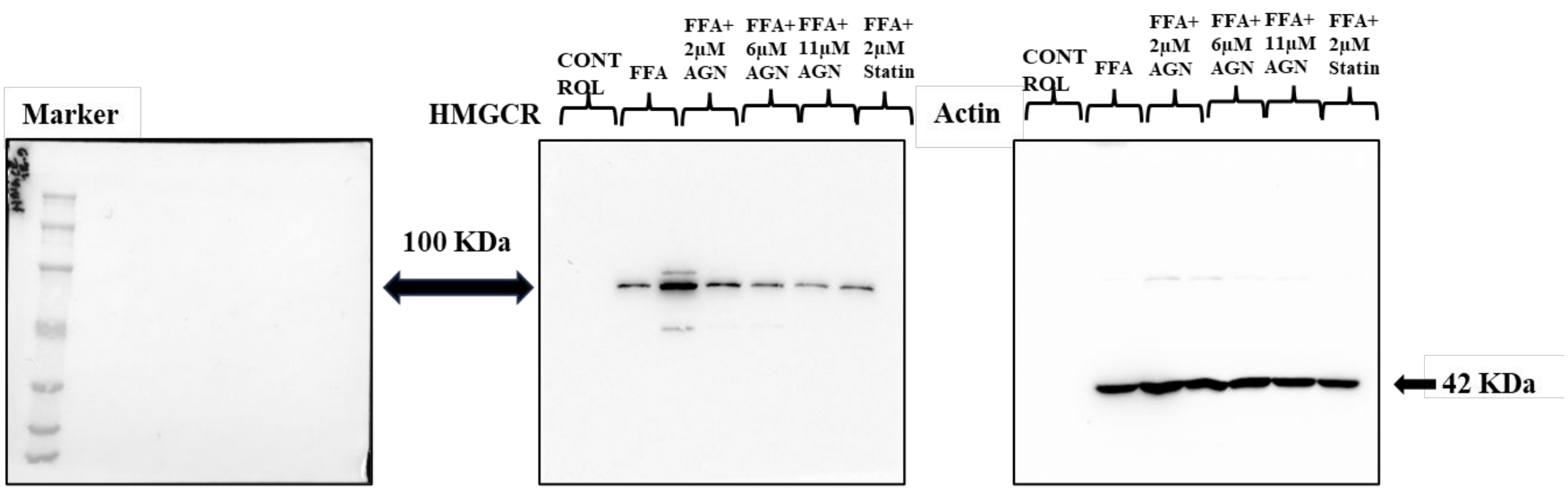

(D) pHMGCR

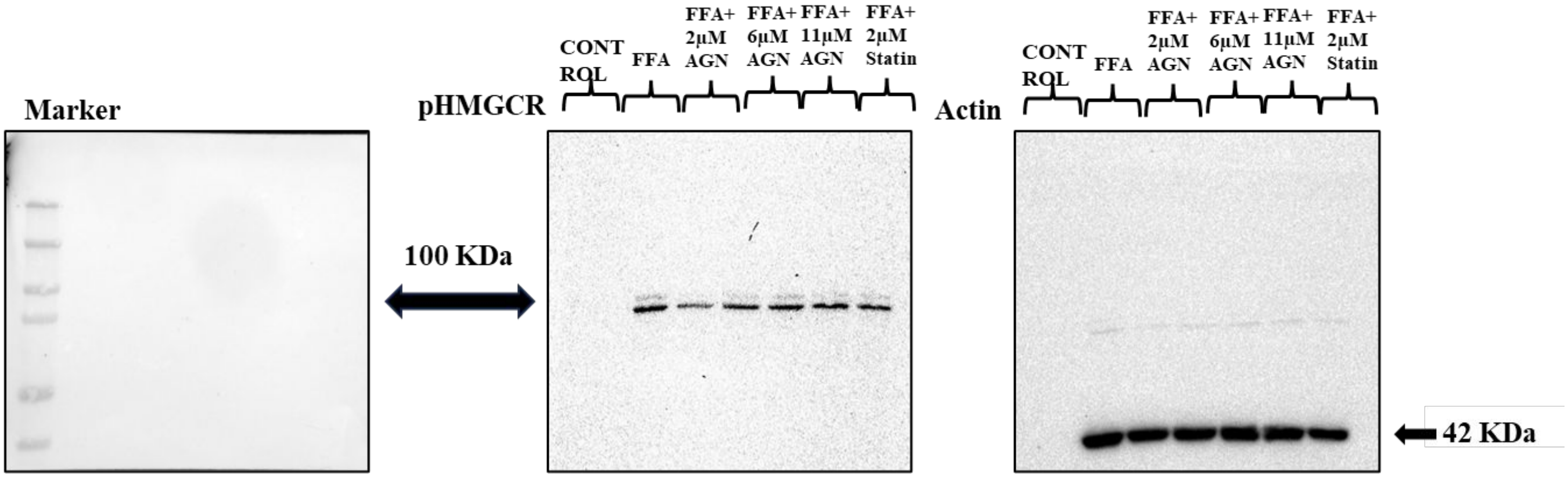

(E) SREBP1C

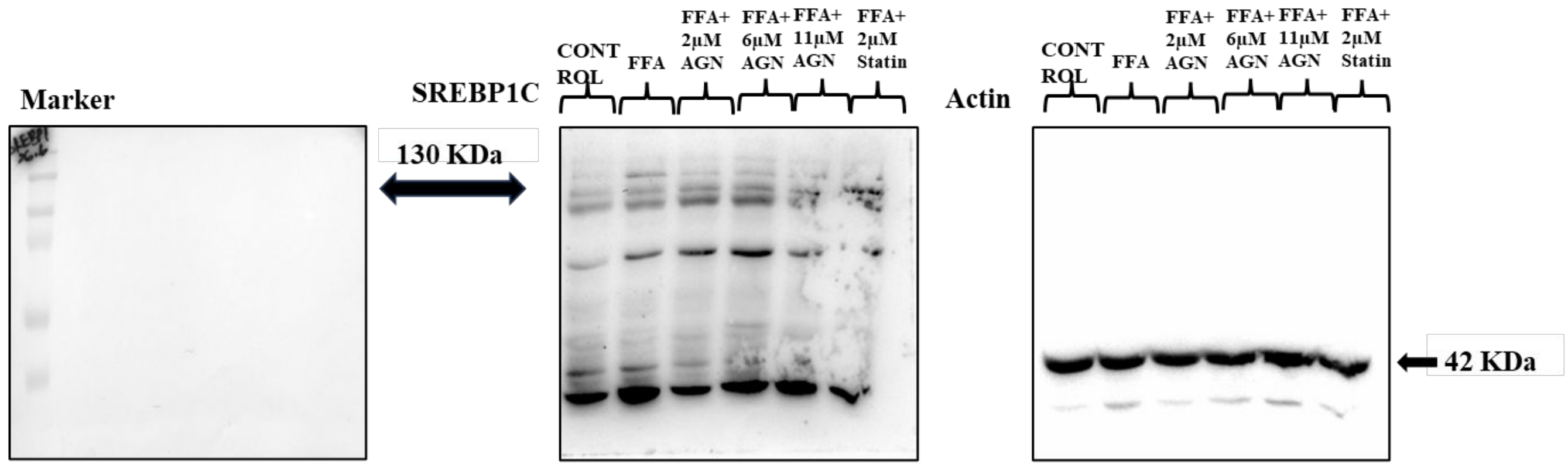

(F) FASN

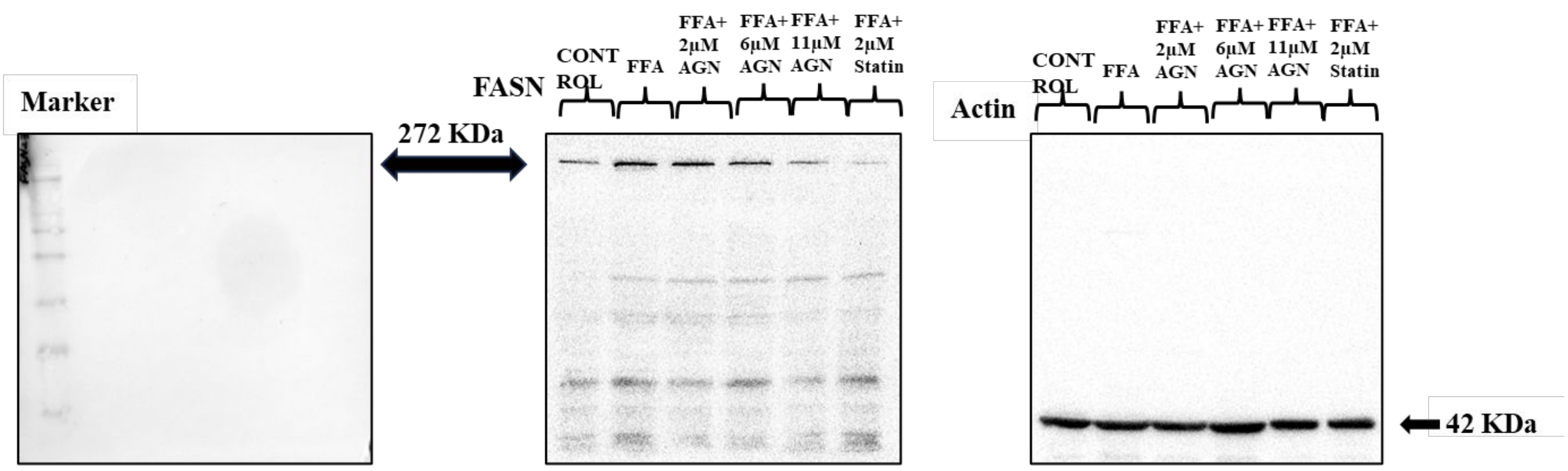

(G) PPARα

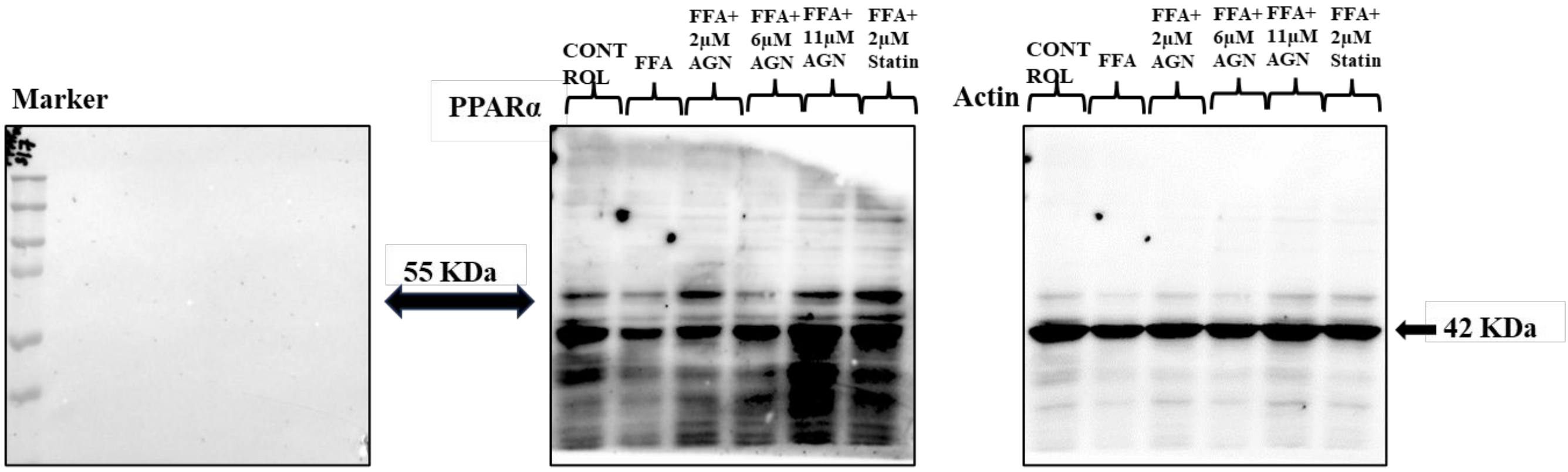

(H) UCP2

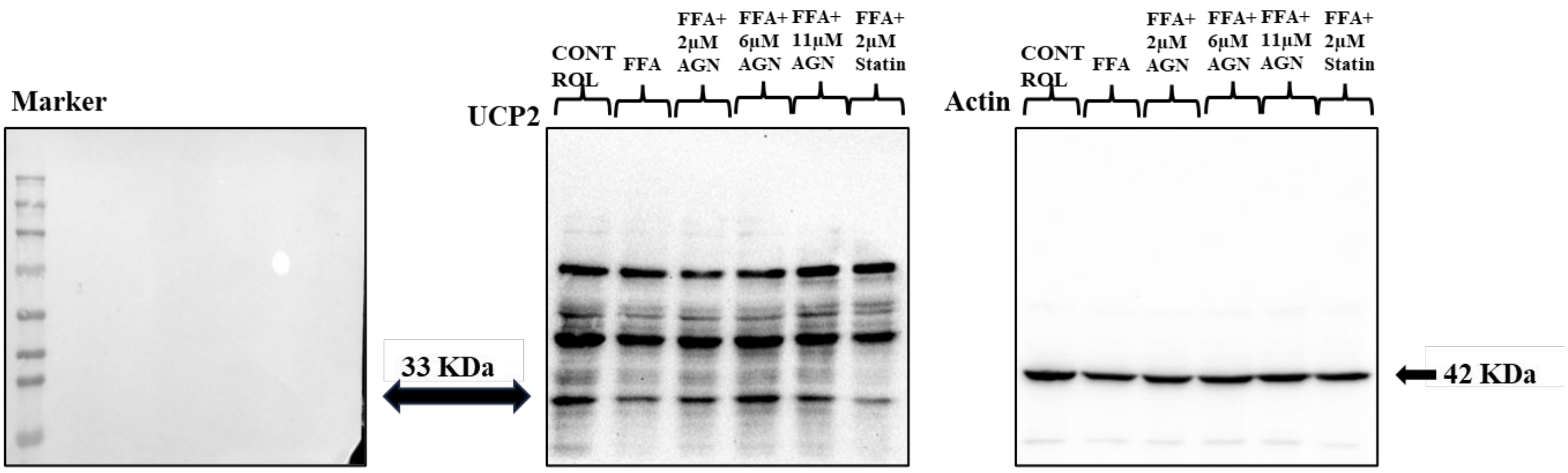

Supplementary Fig 4: RADAR plot showing drug likeliness of AGN, represented by values of different chemical descriptors and their acceptable upper and lower limit values.

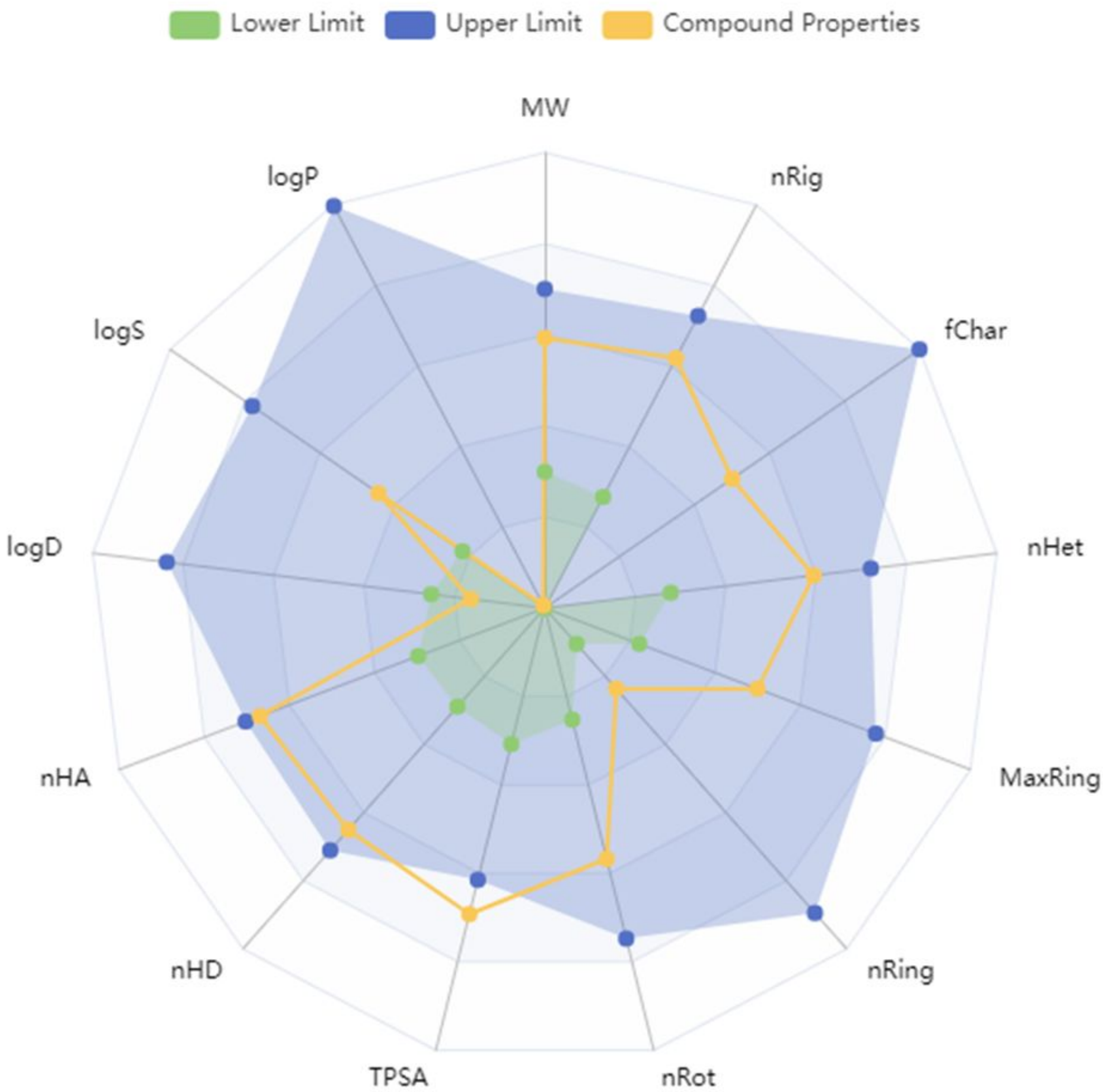

Supplementary Table S1. Predicted ADMET classification parameters for agnuside with probabilities of their occurrence.

Physicochemical Property

Property	Value	Comment
Molecular Weight	466.15	Contain hydrogen atoms. Optimal:100~600
Volume	435.716	Van der Waals volume
Density	1.07	Density = MW / Volume
nHA	11.0	Number of hydrogen bond acceptors. Optimal:0~12
nHD	6.0	Number of hydrogen bond donors. Optimal:0~7
nRot	7.0	Number of rotatable bonds. Optimal:0~11
nRing	1.0	Number of rings. Optimal:0~6
MaxRing	9.0	Number of atoms in the biggest ring. Optimal:0~18
nHet	11.0	Number of heteroatoms. Optimal:1~15
fChar	0.0	Formal charge. Optimal:-4 ~4
nRig	23.0	Number of rigid bonds. Optimal:0~30
Flexibility	0.304	Flexibility = nRot /nRig
Stereo Centers	9.0	Stereo Centers. Optimal: ≤ 2
TPSA	175.37	Topological Polar Surface Area. Optimal:0~140
logS	-2.199	The logarithm of aqueous solubility value.
logP	0.011	The logarithm of the n-octanol/water distribution coefficients at pH=7.4.
logD	0.7	The logarithm of the n-octanol/water distribution coefficient.
pka (Acid)	6.894	Acid-base dissociation constant (pKa) value represents the strength of a drug molecule's acidity or basicity.
pka (Base)	4.98	Acid-base dissociation constant (pKa) value represents the strength of a drug molecule's acidity or basicity.
Melting point	183.189	The predicted melting point of a compound is expressed in degrees Celsius (°C).<br>Melting points below 25°C are classified as liquids, while melting points above 25°C are classified as solids.

Boiling point	335.413	The predicted melting point of a compound is expressed in degrees Celsius (°C).<br>A normal boiling point below 25°C is categorized as a gas.

Medicinal Chemistry

Property	Value	Decision	Comment
QED	0.219	🔴	☐ A measure of drug-likeness based on the concept of desirability;<br>☐ Attractive: > 0.67;<br>☐ unattractive: 0.49~0.67;<br>☐ too complex: < 0.34
GASA	1.0	🔴	☐ ES: Easy to synthesize; HS: Hard to synthesize;<br>☐ The output value represents the probability of being difficult to synthesize, ranging from 0 to 1.
Synth	4.0	🟢	☐ Synthetic accessibility score is designed to estimate ease of synthesis of drug-like molecules.<br>☐ SAscore ≥ 6, difficult to synthesize; SAscore <6 easy to synthesize
Fsp3	0.5	🟢	☐ The number of sp3 hybridized carbons / carbon count, correlating with melting point and solubility.<br>☐ Fsp^3 ≥0.42 is considered a suitable value.
MCE-18	88.636	🟢	☐ MCE-18 stands for medicinal chemistry evolution.<br>☐ MCE-18≥45 is considered a suitable value.
NPscore	2.234	-	☐ Natural product-likeness score.<br>☐ This score is typically in the range from -5 to 5.<br>☐ The higher the score is, the higher the probability is that the molecule is a NP.
Lipinski Rule	1.0	🔴	☐ MW ≤ 500; log P ≤ 5; Hacc ≤ 10; Hdon ≤ 5<br>☐ If two properties are out of range, a poor absorption or permeability is possible, one is acceptable.

Pfizer Rule	0.0	●	□ logP > 3; TPSA < 75<br>□ Compounds with a high log P (>3) and low TPSA (<75) are likely to be toxic.
GSK Rule	1.0	●	□ MW ≤ 400; logP ≤ 4<br>□ Compounds satisfying the GSK rule may have amore favorable ADMET profile
Golden Triangle	0.0	●	□ 200 ≤ MW ≤ 500; -2 ≤ logD ≤ 5<br>□ Compounds satisfying the Golden Triangle rule may have a more favorable ADMET profile.
PAINS	0 alerts	-	frequent hitters, Alpha-screen artifacts and reactive compound 480 substructures (J Med Chem 201053:2719-40)
ALARM NMR	2 alerts	-	Thiol reactive compounds.
BMS	0 alerts	-	undesirable, reactive compounds 176 substructures (J Chem Inf Model 200646:1060-8)
Chelator Rule	0 alerts	-	Chelating compounds.
Colloidal aggregators	0.567	-	□ Category 0: non-colloidal aggregators;<br>□ Category 1: colloidal aggregators.<br>□ The output value is the probability of being colloidal aggregators, within the range of 0 to 1.
FLuc inhibitors	0.221	●	□ Category 0: non-fLuc inhibitors;<br>□ Category 1: fLuc inhibitors.<br>□ The output value is the probability of being fLuc inhibitors, within the range of 0 to 1.
Blue fluorescence	0.178	●	□ Category 0: non-blue fluorescence;<br>□ Category 1: blue fluorescence.<br>□ The output value is the probability of being blue fluorescence, within the range of 0 to 1.
Green fluorescence	0.055	●	□ Category 0: non-green fluorescence;<br>□ Category 1: green fluorescence.<br>□ The output value is the probability of being green fluorescence, within the range of 0 to 1.
Reactive compounds	0.247	●	□ Category 0: non-reactive compound;<br>□ Category 1: reactive compound.<br>□ The output value is the probability of being reactive compound, within the range of 0 to 1.

Promiscuous compounds	0.335	●	□ Category 0: non-promiscuous compound;<br>□ Category 1: promiscuous compound.<br>□ The output value is the probability of being promiscuous compound, within the range of 0 to 1.

Absorption

**Property**	**Value**	**Decision**	**Comment**
Caco-2 Permeability	-6.328	●	Optimal: higher than -5.15 Log unit
MDCK Permeability	-5.16	●	□ low permeability: < 2 × 10-6 cm/s<br>□ medium permeability: 2-20 × 10^{-6} cm/s<br>□ high passive permeability: > 20 × 10^{-6} cm/s
PAMPA	0.989	●	□ The experimental data for Peff was logarithmically transformed (logPeff).<br>□ Molecules with log Peff values below 2.0 classified as low-permeability (Category 0), while those with log Peff values exceeding 2.5 were classified as high-permeability (Category 1).
Pgp-inhibitor	0.0	●	□ Category 1: Inhibitor;<br>□ Category 0: Non-inhibitor;<br>□ The output value is the probability of being Pgp-inhibitor
Pgp-substrate	0.48	●	□ Category 1: substrate;<br>□ Category 0: Non-substrate;<br>□ The output value is the probability of being Pgp-substrate
HIA	0.238	●	□ Human Intestinal Absorption<br>□ Category 1: HIA+(HIA < 30%);<br>□ Category 0: HIA-(HIA >= 30%);<br>□ The output value is the probability of being HIA+
$F_{20\%}$	0.532	●	□ 20% Bioavailability<br>□ Category 1: F 20% + (bioavailability < 20%);<br>□ Category 0: F 20% - (bioavailability ≥ 20%);<br>□ The output value is the probability of being F 20%<br>+

$F_{30\%}$	0.995	●	□ 30% Bioavailability<br>□ Category 1: F 30% + (bioavailability < 30%);<br>□ Category 0: F 30% - (bioavailability ≥ 30%);<br>□ The output value is the probability of being F 30%<br>+
$F_{50\%}$	0.982	●	□ 50% Bioavailability<br>□ Category 1: F 50% + (bioavailability < 50%);<br>□ Category 0: F 50% - (bioavailability ≥ 50%);<br>□ The output value is the probability of being F 50%<br>+

Distribution

Property	Value	Decision	Comment
PPB	74.029	●	□ Plasma Protein Binding Optimal: < 90%.<br>□ Drugs with high protein-bound may have a low therapeutic index.
VDss	-0.336	●	□ Volume Distribution<br>□ Optimal: 0.04-20L/kg
BBB	0.005	●	□ Blood-Brain Barrier Penetration<br>□ Category 1: BBB+; Category 0: BBB-;<br>□ The output value is the probability of being BBB+
Fu	27.96	●	□ The fraction unbound in plasms<br>□ Low: <5%; Middle: 5~20%; High: > 20%
OATP1B1 inhibitor	0.993	●	□ Category 0: Non-inhibitor; Category 1: inhibitor.<br>□ The output value is the probability of being inhibitor, within the range of 0 to 1.
OATP1B3 inhibitor	0.997	●	□ Category 0: Non-inhibitor; Category 1: inhibitor.<br>□ The output value is the probability of being inhibitor, within the range of 0 to 1.
BCRP inhibitor	0.028	●	□ Category 0: Non-inhibitor; Category 1: inhibitor.<br>□ The output value is the probability of being inhibitor, within the range of 0 to 1.

MRP1 inhibitor	0.122	●	□ Category 0: Non-inhibitor; Category 1: inhibitor.<br>□ The output value is the probability of being inhibitor, within the range of 0 to 1.

Metabolism

Property	Value	Decision	Comment
CYP1A2 inhibitor	0.0	●	☐ Category 1: Inhibitor; Category 0: Non-inhibitor; ☐ The output value is the probability of being inhibitor.
CYP1A2 substrate	0.0	●	☐ Category 1: Substrate; Category 0: Non-substrate; ☐ The output value is the probability of being substrate.
CYP2C19 inhibitor	0.0	●	☐ Category 1: Inhibitor; Category 0: Non-inhibitor; ☐ The output value is the probability of being inhibitor.
CYP2C19 substrate	0.0	●	☐ Category 1: Substrate; Category 0: Non-substrate; ☐ The output value is the probability of being substrate.
CYP2C9 inhibitor	0.0	●	☐ Category 1: Inhibitor; Category 0: Non-inhibitor; ☐ The output value is the probability of being inhibitor.
CYP2C9 substrate	0.0	●	☐ Category 1: Substrate; Category 0: Non-substrate; ☐ The output value is the probability of being substrate.
CYP2D6 inhibitor	0.0	●	☐ Category 1: Inhibitor; Category 0: Non-inhibitor; ☐ The output value is the probability of being inhibitor.
CYP2D6 substrate	0.0	●	☐ Category 1: Substrate; Category 0: Non-substrate; ☐ The output value is the probability of being substrate.
CYP3A4 inhibitor	0.006	●	☐ Category 1: Inhibitor; Category 0: Non-inhibitor; ☐ The output value is the probability of being inhibitor.
CYP3A4 substrate	0.0	●	☐ Category 1: Substrate; Category 0: Non-substrate; ☐ The output value is the probability of being substrate.

CYP2B6 inhibitor	0.0	●	☐ Category 1: Inhibitor; Category 0: Non-inhibitor;<br>☐ The output value is the probability of being inhibitor.
CYP2B6 substrate	0.0	●	☐ Category 1: Substrate; Category 0: Non-substrate;<br>☐ The output value is the probability of being substrate.
CYP2C8 inhibitor	0.66	●	☐ Category 1: Inhibitor; Category 0: Non-inhibitor;<br>☐ The output value is the probability of being inhibitor.
HLM Stability	0.005	●	☐ human liver microsomal (HLM) stability<br>☐ Category 0: stable+ (HLM > 30 min); Category 1: unstable- (HLM ≤ 30 min). The output value is the probability of human liver microsomal instability, where a value closer to 1 indicates a higher likelihood of instability. The range is between 0 and 1.

Excretion

Property	Value	Decision	Comment
CL_{plasma}	2.958	●	☐ The unit of predicted CLplasma penetration is ml/min/kg. >15 ml/min/kg: high clearance; 5-15 ml/min/kg: moderate clearance; < 5 ml/min/kg: low clearance.
$T_{1/2}$	2.813	●	☐ The unit of predicted T1/2 is hours.<br>☐ ultra-short half-life drugs: 1/2 < 1 hour; short half-life drugs: T1/2 between 1-4 hours; intermediate short half-life drugs: T1/2 between 4-8 hours; long half-life drugs: T1/2 > 8 hours.

Toxicity

Property	Value	Decision	Comment
hERG Blockers	0.031	●	☐ Molecules with IC50 ≤10μM or ≥50% inhibition at 10 μM were classified as hERG+ (Category 1),<br>☐ while molecules with IC50 >10μM or < 50% inhibition at 10μM were classified as hERG - (Category 0).<br>☐ The output value is the probability of being

			hERG+, within the range of 0 to 1.
hERG Blockers (10um)	0.227	●	☐ Molecules with IC50 ≤10 µM are classified as hERG+ (Category 1),<br>☐ and molecules with IC50 > 10 µM are classified hERG - (Category 0).<br>☐ The output value is the probability of being hERG+, within the range of 0 to 1.
DILI	0.877	●	☐ Drug-Induced Liver Injury.<br>☐ Category 1: drugs with a high risk of DILI;<br>☐ Category 0: drugs with no risk of DILI.<br>☐ The output value is the probability of being toxic.
AMES Mutagenicity	0.836	●	☐ AMES Toxicity<br>☐ Category 1: Ames positive(+);<br>☐ Category 0: Ames negative(-);<br>☐ The output value is the probability of being toxic.
Rat Oral Acute Toxicity	0.014	●	☐ Rat Oral Acute Toxicity.<br>☐ Category 0: low-toxicity, > 500 mg/kg;<br>☐ Category 1: high toxicity; < 500 mg/kg.<br>☐ The output value is the probability of being toxic, within the range of 0 to 1.
FDAMDD	0.032	●	☐ FDA Maximum (Recommended) Daily Dose.<br>☐ Category 1: FDAMDD (+);<br>☐ Category 0: FDAMDD (-);<br>The output value is the probability of being positive.
Skin Sensitization	0.996	●	☐ Category 1: Sensitizer;<br>☐ Category 0: Non-sensitizer.<br>☐ The output value is the probability of being toxic, within the range of 0 to 1.
Carcinogenicity	0.479	●	☐ Category 1: carcinogens;<br>☐ Category 0: non-carcinogens;<br>☐ The output value is the probability of being toxic.
Eye Corrosion	0.0	●	☐ Eye Corrosion<br>☐ Category 1: corrosives; Category 0: noncorrosive;<br>☐ The output value is the probability of being corrosive.

Eye Irritation	0.234	●	□ Eye Irritation<br>□ Category 1: irritants; Category 0: nonirritants;<br>□ The output value is the probability of being irritant.
Respiratory	0.004	●	□ Category 1: respiratory toxicants;<br>□ Category 0: non-respiratory toxicants.<br>□ The output value is the probability of being toxic, within the range of 0 to 1.
Human Hepatotoxicity	0.146	●	□ Human Hepatotoxicity<br>□ Category 1: H-HT positive(+);<br>□ Category 0: H-HT negative(-);<br>□ The output value is the probability of being toxic.
Drug-induced Nephrotoxicity	0.477	●	□ Category 0: non-nephrotoxic (-);<br>□ Category 1: nephrotoxic (+).<br>□ The output value is the probability of being nephrotoxic (+), within the range of 0 to 1.
Ototoxicity	0.92	●	□ Category 0: non-ototoxicity (-);<br>□ Category 1: ototoxicity (+).<br>□ The output value is the probability of being ototoxicity (+)within the range of 0 to 1.
Hematotoxicity	0.039	●	□ Category 0: non-hematotoxicity (-);<br>□ Category 1: hematotoxicity (+).<br>□ The output value is the probability of being hematotoxicity (+), within the range of 0 to 1.
Genotoxicity	0.263	●	□ Category 0: non-Genotoxicity (-);<br>□ Category 1: Genotoxicity (+).<br>□ The output value is the probability of being ototoxicity (+), within the range of 0 to 1.
RPMI-8226 Immunitoxicity	0.126	●	□ Category 0: non-cytotoxicity (-);<br>□ Category 1: cytotoxicity (+).<br>□ The output value is the probability of being ototoxicity (+), within the range of 0 to 1.
A549 Cytotoxicity	0.293	●	□ Category 0: non-cytotoxicity (-);<br>□ Category 1: cytotoxicity (+).<br>□ The output value is the probability of being ototoxicity (+)within the range of 0 to 1.

Hek293 Cytotoxicity	0.415	●	□ Category 0: non-cytotoxicity (-);<br>□ Category 1: cytotoxicity (+).<br>□ The output value is the probability of being ototoxicity (+)within the range of 0 to 1.
Drug-induce d Neurotoxi city	0.025	●	□ Category 0: non-neurotoxic (-);<br>□ Category 1: neurotoxic (+).<br>□ The output value is the probability of being neurotoxic (+)within the range of 0 to 1.

Environmental toxicity

Property	Value	Comment
Bioconcentration Factors	0.382	□ Bioconcentration factors are used to consider secondary poisoning potential and assess risks to human health via the food chain.<br>□ The unit is -log10[(mg/L)/(1000*MW)]
IGC_{50}	3.104	□ Tetrahymena pyriformis has a 50 percent growth inhibition concentration.<br>□ The unit is -log10[(mg/L)/(1000*MW)]
$LC_{50}FM$	3.709	□ 96-hour fathead minnow 50 per cent lethal concentration.<br>□ The unit is -log10[(mg/L)/(1000*MW)]
$LC_{50}DM$	4.63	□ 48-hour daphnia magna 50 per cent lethal concentration.<br>□ The unit is -log10[(mg/L)/(1000*MW)]

Tox21 pathway

Property	Value	Decision	Comment
NR-AhR	0.055	●	□ Aryl hydrocarbon receptor<br>□ Category 1: actives ;<br>□ Category 0: inactive;<br>□ The output value is the probability of being active.
NR-AR	0.014	●	□ Androgen receptor<br>□ Category 1: actives ;<br>□ Category 0: inactive;<br>□ The output value is the probability of being active.

NR-AR-LBD	0.001		□ Androgen receptor ligand-binding domain<br>□ Category 1: actives ;<br>□ Category 0: inactive;<br>□ The output value is the probability of being active.
NR-Aromatase	0.125		□ Category 1: actives ;<br>□ Category 0: inactive;<br>□ The output value is the probability of being active.
NR-ER	0.99		□ Estrogen receptor<br>□ Category 1: actives ;<br>□ Category 0: inactive;<br>□ The output value is the probability of being active.
NR-ER-LBD	0.529		□ Estrogen receptor ligand-binding domain<br>□ Category 1: actives ;<br>□ Category 0: inactive;<br>□ The output value is the probability of being active.
NR-PPAR-gamma	0.0		□ Peroxisome proliferator-activated receptor gamma<br>□ Category 1: actives ;<br>□ Category 0: inactive;<br>□ The output value is the probability of being active.
SR-ARE	0.04		□ Antioxidant response element<br>□ Category 1: actives ;<br>□ Category 0: inactive;<br>□ The output value is the probability of being active.

SR-ATAD5	0.002		□ ATPase family AAA domain-containing protein 5<br>□ Category 1: actives ;<br>□ Category 0: inactive;<br>□ The output value is the probability of being active.
SR-HSE	0.001		□ Heat shock factor response element<br>□ Category 1: actives ;<br>□ Category 0: inactive;<br>□ The output value is the probability of being active.
SR-MMP	0.626		□ Mitochondrial membrane potential<br>□ Category 1: actives ;<br>□ Category 0: inactive;<br>□ The output value is the probability of being

			active.
SR-p53	0.016	●	- p53, a tumor suppressor protein<br>- Category 1: actives ;<br>- Category 0: inactive;<br>- The output value is the probability of being active.

Toxicophore Rules

Property	Value	Comment
Acute Toxicity Rule	0	- 20 substructures;<br>- acute toxicity during oral administration
Genotoxic Carcinogenicity Rule	3 alerts	- 117 substructures;<br>- carcinogenicity or mutagenicity
NonGenotoxic Carcinogenicity Rule	0	- 23 substructures;<br>- carcinogenicity through nongenotoxic mechanisms
Skin Sensitization Rule	2 alerts	- 155 substructures;<br>- skin irritation
Aquatic Toxicity Rule	2 alerts	- 99 substructures;<br>- toxicity to liquid(water)
NonBiodegradable Rule	1 alerts	- 19 substructures;<br>- non-biodegradable
SureChEMBL Rule	0	- 164 substructures;<br>- MedChem unfriendly status
Toxicophores Rule	1 alerts	154 toxic substructures from FAF-Drug4

Supplementary Figure 05: HMG-CoA Reductase Inhibition Assay: IC_{50} was calculated by interpolating the concentration vs. % inhibition curve (best fiting line) in GraphPad Prism version 5, setting Force 0 (X=0; Y=0). Vales are presented as MEAN±SEM (n=03).

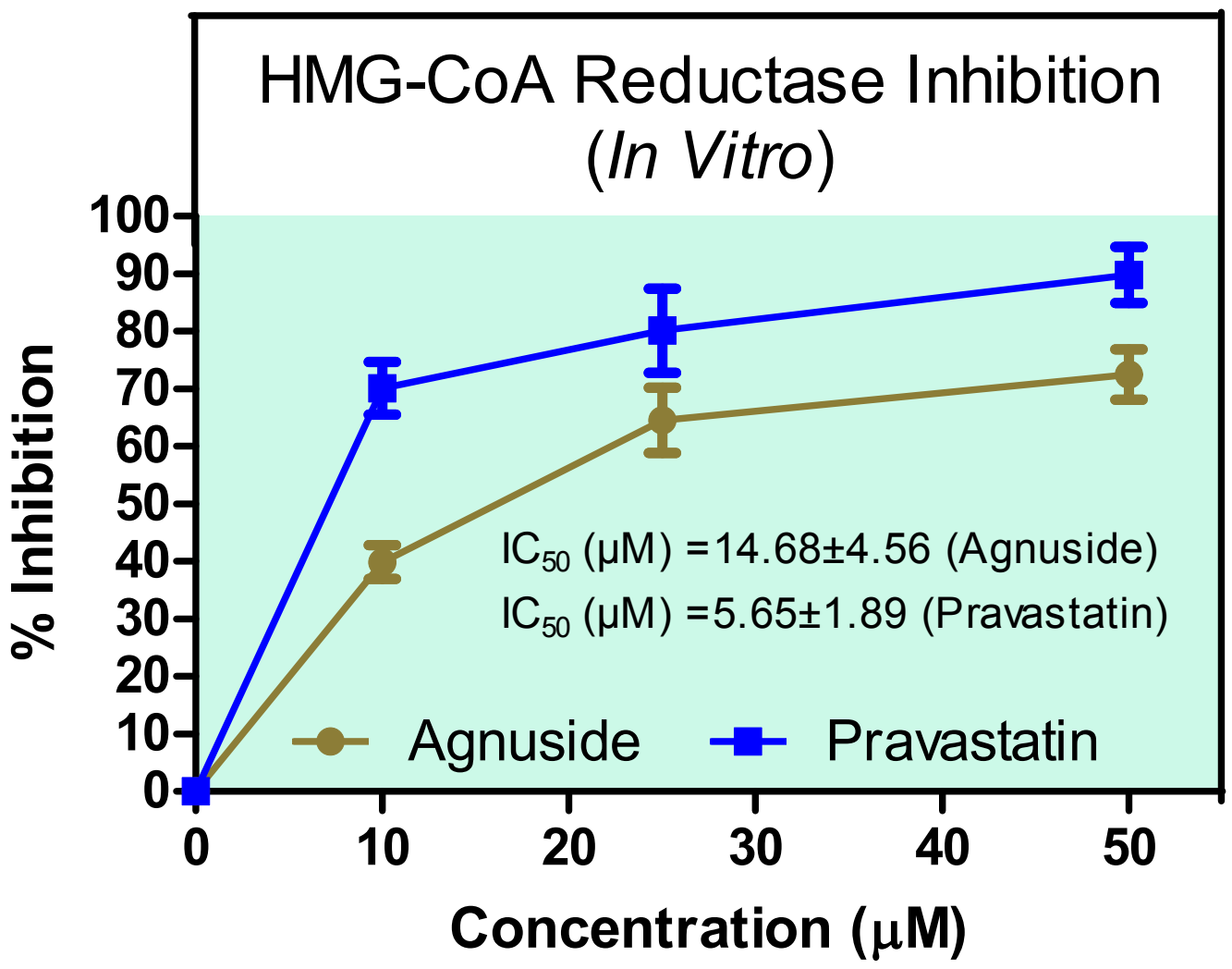

Supplement: Supplementary file 1 [file ao6c06583_si_001.pdf]
